# The efficacy and safety of immunosuppressive therapies in the treatment of IgA nephropathy: A network meta-analysis

**DOI:** 10.1038/s41598-020-63170-w

**Published:** 2020-04-08

**Authors:** Jiaxing Tan, Lingqiu Dong, Donghui Ye, Yi Tang, Tengyue Hu, Zhengxia Zhong, Padamata Tarun, Yicong Xu, Wei Qin

**Affiliations:** 1 Division of Nephrology, Department of Medicine, West China Hospital, Sichuan University, Chengdu, Sichuan China; 2 West China School of Medicine, Sichuan University, Chengdu, Sichuan China

**Keywords:** Glomerular diseases, Nephritis

## Abstract

Immunoglobulin A nephropathy (IgAN) is a common autoimmune glomerulonephritis that can result in end-stage renal disease (ESRD). Whether immunosuppressants are superior or equivalent to supportive care is still controversial. A network meta-analysis was conducted to compare the efficacy and safety of immunosuppressive treatment for IgAN. Medline, Cochrane Central Register of Controlled Trials (CENTRAL), Web of Science, and EMBASE were searched on December 30, 2018. We used a random-effects model with a Bayesian approach to appraise both renal outcomes and serious adverse effects. Relative risks (RRs) with 95% confidence intervals (CIs) were calculated to present the relative effects. The ranking probabilities were calculated by the surface under the cumulative ranking curve (SUCRA). In total, 24 RCTs comprising 6 interventions were analyzed. Steroids significantly delayed the progression of renal deterioration with acceptable serious adverse effects, compared with supportive care (RR = 0.28, 95% CI = 0.13–0.51, SUCRA = 48.7%). AZA combined with steroids might be an alternative immunosuppressive therapy. Tacrolimus might decrease the proteinuria level (RR = 3.1, 95% CI = 1.2–9.4, SUCRA = 66.5%) but cannot improve renal function, and the side effects of tacrolimus should not be neglected. MMF and CYC showed no superiority in the treatment of IgAN. In summary, steroids might be recommended as the first-line immunosuppressive therapy for IgAN.

## Introduction

Immunoglobulin A nephropathy (IgAN), characterized by diffuse IgA deposits in the mesangial glomeruli with or without deposition of other immunoglobulins, is one of the most common kidney diseases in the world^1^. IgAN is manifested by recurrent hematuria and/or proteinuria, which was initially regarded as a benign disease^2^. As research has advanced, it has been found that the natural course of IgAN is far from benign, and severe deterioration of renal function may occur. Approximately 20–40% of patients with IgAN will progress to end-stage renal disease (ESRD) or need continuous renal replacement therapies within 10–20 years^3^. Consequently, finding an optimal strategy that prevents renal failure in patients is of great importance.

It is well acknowledged that IgAN is an autoimmune disease, suggesting that immunosuppressive treatment may potentially contribute to clinical remission^4^. Currently, there are 5 immunosuppressants that are commonly used for patients with IgAN in the clinic: steroids, tacrolimus (TAC), cyclophosphamide (CYC), mycophenolate mofetil (MMF), and azathioprine (AZA). However, the efficacy and safety of these immunosuppressants in treating IgAN are under debate. A previous pairwise meta-analysis proposed that immunosuppressive agents were a superior option, but it considered only a proteinuria decrease and did not investigate the effects of the immunosuppressants on the prevention of renal deterioration. In addition, this study did not investigate which immunosuppressive therapies were the best options for IgAN^5^. Therefore, its findings have not been widely accepted. Moreover, only two therapeutic regimens could be analyzed by the pairwise meta-analysis, and therefore, the superiority of each immunosuppressive agent has not yet been elucidated. Whether immunosuppressants are superior or equivalent to supportive care is still controversial due to the limited direct comparative evidence. For this reason, a systematic review and network meta-analysis, which can compare all drug classes simultaneously, was undertaken to indirectly assess the first-line immunosuppressive treatments of IgAN.

## Methods

The protocol of this systematic review and network meta-analysis was submitted to the PROSPERO register and the registration number is CRD42019122324. The original data are available in the supplementary information. Because no human beings or animals were part of this study, ethics committee approval was not required.

### Search strategies

Two investigators (TJX and DLQ) independently performed a systematic literature retrieval. Commonly used databases, including Medline, Cochrane Central Register of Controlled Trials (CENTRAL), Web of Science, and EMBASE, were searched on December 30, 2018, and the last searched date was April 1, 2019. The text-word terms and subject headings we used in this study were “Immunoglobulin A nephropathy”, “cyclophosphamide”, “azathioprine”, “tacrolimus”, “mycophenolic acid”, “mycophenolate mofetil”, “steroids”, and “glucocorticoid”. The syntax used in each database is shown in Supplementary Table 1 (Table S1). To avoid omitting important articles, we also hand-searched the references of each retrieved study, relevant reviews, editorials and commentary.

### Inclusion and exclusion criteria

Studies matching the following conditions were included. (a) The experimental design was a randomized controlled trial (RCT) on the treatment of IgAN. (b) The intervention plans included steroids, AZA, CYC, MMF, and TAC. (c) The renal outcome data were available. (d) If a cohort was reported more than once, only the latest cohort or the largest cohort was included.

The exclusion criteria in this network meta-analysis were as follows: (a) use of other immunosuppressants, such as traditional Chinese medicine, whose contents were uncertain and might have unknown additional effects; (b) observational studies, editorials, reviews, case reports, comments and other non-RCTs; and (c) a lack of definitions of renal outcomes or clinical remission in the study.

### Study selection

Based on the strict inclusion and exclusion criteria, two authors (TJX and DLQ) evaluated the titles and abstracts independently for the preliminary screening. We performed a full-text review for all the studies that met the requirements. Any discrepancies were settled by discussion or consultation with the third author (YDH).

### Measurement

Primary outcomes were composite endpoints of ESRD, estimated glomerular filtration rate (e-GFR) < 40% of baseline, a 50% increase in serum creatinine (sCr) and/or death. Secondary outcomes were defined as the remission of proteinuria without obvious renal function damage, including complete remission, partial remission and total remission. Complete remission was defined as proteinuria <0.4 g/d. Partial remission was defined as 50% proteinuria reduction or proteinuria ≥0.4 but <1.0 g/24 h. Total remission covered both complete remission and partial remission.Serious adverse events included severe infection, new diabetes, hemorrhage, cardiovascular events, malignancy, osteonecrosis or all-cause mortality.

### Quality assessment

The Cochrane Collaboration’s tool was used to assess the quality of RCTs. This tool included random sequence generation, allocation concealment, blinding of participants, personnel and outcome assessment, incomplete outcome data management and selective reporting to assess the selection bias, performance bias, detection bias, attrition bias, reporting bias and other bias^6^.

The Grading of Recommendations Assessment, Development and Evaluation (GRADE) approach was used to evaluate the confidence in estimates. We assessed the quality of evidence by the four-level scale including high, moderate, low, and very low confidence. A direct comparison was regarded as high quality, which could be rated down if there were study limitations, inconsistency, indirectness, imprecision, and/or publication bias. For indirect estimates, we graded the evidence quality of each comparison which contributed as first-order loops. Then, the lower level was chosen but could be downgraded further for intransitivity or imprecision. If the estimates were similar between direct and indirect comparisons, the higher one was assigned.

We appraised the transitivity assumption of network meta-analysis by analyzing whether there were significant differences in baseline characteristics, common control and result measurement among different groups. The distribution of clinical and methodological variables was fully considered.

### Statistical analysis

This network meta-analysis was carried out by a random-effects model with a Bayesian approach. All direct and indirect comparisons were analyzed by WinBUGS, OpenBUGS, R software and STATA. The “Gemtc” package and JAGS in R were also used. Before quantitative analysis, we drew a network diagram to illustrate the comparisons of treatment regimens in different studies. For each outcome, relative risks (RRs) and their 95% confidence intervals (CIs) were calculated to present the relative effects. To estimate the efficacy of various immunosuppressive therapies, ranking probabilities were determined. Notably, the ranking probabilities were calculated by the surface under the cumulative ranking curve (SUCRA), and a larger SUCRA demonstrated a higher rank of the protocol^7^. Heterogeneity and sensitivity analyses were conducted to verify the reliability of the model. We evaluated inconsistency locally and globally and assessed inconsistency in the entire network by carrying out the sidesplitting method and the loop-specific approach, and fitting the design by treatment.

## Results

### Selection and characteristics of the studies

**Figure 1:**
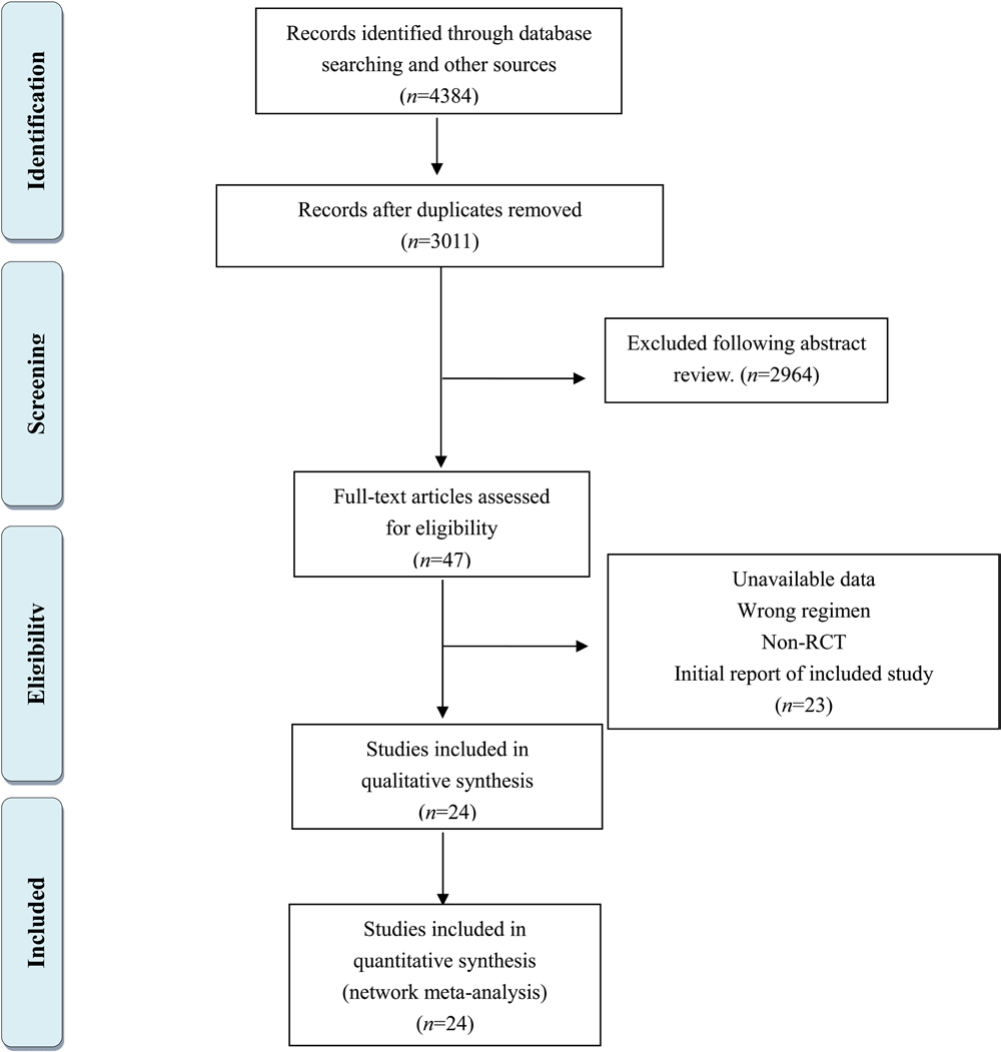
Flowchart illustrating the selection of studies.

A total of 3,011 unique articles collected from different databases were identified after removing duplicates. Based on the strict criteria, 2,964 references were removed through an abstract search (Fig. 1). Afterwards, the full texts of 42 studies were further screened, and the results are provided in Table S2. In total, 24 RCTs involving 25 direct comparisons were analyzed qualitatively and quantitatively^8^. Table 1 reveals the characteristics of all selected studies. Seven interventions, including supportive care, steroids, CYC, TAC, MMF, AZA and CYC + AZA were compared in the studies. For these RCTs, 11 compared steroids with supportive care^8–18^. Four studies compared MMF with supportive care^19–22^. One study compared MMF plus steroids with steroids^23^. One compared MMF plus steroids with CYC plus steroids^24^. MMF monotherapy and MMF plus steroids were regarded as the same group (MMF group). Two studies investigated CYC and prednisolone for the initial 3 months, then azathioprine at the same dose continued for a minimum of 2 years^8,25^. Two studies compared AZA plus steroids with supportive care^26,27^. Two studies compared AZA plus steroids with steroids^28,29^. Two compared TAC monotherapy with supportive care^30,31^. Of the 24 RCTs, 11 studies used ACEI/ARB in all participants. ACEI/ARB was used in another 8 studies, but the percentages could not be calculated. The remaining studies did not include ACEI/ARB. In total, 2,000 patients with IgAN were included, and the results of the quality assessment are illustrated in Table S3.

**Table 1:** Characteristics of immunosuppressive therapies for IgA nephropathy included in the network meta-analysis.

Study	Patients	Regimens		Sample size		Age		Follow-up	
		T	C	T (male)	C (male)	T	C	T	C
Fellstrom 2017	e-GFR > 45 mL/min/1·73 m², proteinuria > 0.75 g/d	S	Sup.	99 (70)	50 (35)	39.0 ± 12.3	39.0 ± 12.3	12 m	12 m
Hogg 2006	e-GFR ≥ 50 mL/min/1·73 m², moderate to severe proteinuria	S	Sup.	33 (23)	31 (20)	24 ± 10	21 ± 10	25 m	25 m
Lv 2017	e-GFR 20 to 120 mL/min/1·73 m², proteinuria > 1 g/d	S	Sup.	136 (86)	126 (80)	38.6 ± 11.5	38.6 ± 10.7	60 m	60 m
Manno 2009	e-GFR ≥ 50 mL/min/1·73 m², proteinuria ≥ 1.0 g/d	S	Sup.	48 (33)	49 (35)	31.8 ± 11.3	34.9 ± 11.2	63.0 m	57.2 m
Pozzi 2004	sCr ≤ 1.5 mg/dL proteinuria, 1.0–3.5 g/d	S	Sup.	43 (30)	43 (31)	38 (26–45)	40 (29–51)	84 m	84 m
Katafuchi 2003	sCr ≤ 1.5 mg/dL	S	Sup.	43 (15)	47 (22)	33.6 ± 13.4	32.5 ± 10.8	25 m	23 m
Lv 2009	e-GFR > 30 mL/min/1·73 m², proteinuria 1.0–5.0 g/d	S	Sup.	33 (20)	30 (19)	27.8 ± 8.9	30.43 ± 8.8	48 m	48 m
Harmankaya 2002	isolated hematuria, and well-preserved renal function	AZA	Sup.	21 (15)	22 (14)	25 (13–42)	27 (17–63)	64 m	58 m
Lai 1986	nephrotic syndrome	S	Sup.	17	17	NA	NA	38 m	38 m
Shoji 2000	sCr ≤ 1.5 mg/dL, proteinuria < 1.5 g/d	S	Sup.	11 (5)	8 (1)	28.7 ± 11.2	33.3 ± 11.9	13.4 m	13.4 m
Julian 1993	sCr clearance > 25 mL/min/1·73 m²	S	Sup.	18	17	NA	NA	NA	NA
Liu 2014	e-GFR > 30 mL/min/1·73 m², proteinuria > 1.0 g/d	MMF	CYC	42 (24)	42 (27)	39.8 ± 3.81	37.4 ± 4.78	30.3 m	26.9 m
Hou 2017	e-GFR > 30 mL/min/1·73 m², proteinuria ≥ 1.0 g/d	MMF	S	86 (39)	88 (38)	30.5 (25–37)	32.5 (25–43)	12 m	12 m
Hogg 2015	e-GFR ≥ 40 mL/min/1·73 m², UPCR > 0.8 g/g	MMF	Sup.	25 (14)	27 (18)	31.8 ± 11.7	32.2 ± 13.2	15 m	15 m
Yu 2017	e-GFR ≥ 40 mL/min/1·73 m², UACR 0.3–3.0 g/g cr	TAC	Sup.	18 (6)	19 (5)	36.8 ± 11.3	41.0 ± 12.6	57.9 m	57.9 m
Kim 2013	e-GFR ≥ 40 mL/min/1·73 m², UACR 0.3–3.0 g/g cr	TAC	Sup.	20 (6)	20 (6)	36.9 ± 11.4	40.1 ± 12.8	4 m	4 m
Frisch 2005	advanced IgAN, and creatinine clearance < 80 ml/min	MMF	Sup.	17 (16)	15 (11)	39 (19–72)	37 (22–59)	14.8 m	18.7 m
Maes 2004	proteinuria > 1.0 g/d	MMF	Sup.	21 (16)	13 (8)	39 ± 11	43 ± 15	36 m	36 m
Rauen 2018	e-GFR ≥ 60 mL/min/1·73 m², proteinuria 0.75–3.5 g/d	S	Sup.	55 (42)	54 (47)	41.7 ± 13.3	45.6 ± 11.9	36 m	36 m
Rauen 2018	e-GFR 30–59 mL/min/1·73 m², proteinuria 0.75–3.5 g/d	CYC + AZA	Sup.	27 (20)	26 (18)	45.1 ± 12.8	46.0 ± 14.0	36 m	36 m
Kamei 2011	e-GFR ≥ 30 mL/min/1·73 m², proteinuria ≥ 1.0 g/d	AZA	Sup.	40 (22)	38 (29)	12.2 ± 3.0	11.6 ± 2.3	138 m	84 m
Pozzi 2010	sCr ≤ 2.0 mg/dL, proteinuria ≥ 1.0 g/d	AZA	S	101 (76)	106 (75)	34.8 (27.7–43.9)	40.5 (30.3–51.3)	58.8 m	58.8 m
Yoshikawa 2006	NA	AZA	S	40 (22)	40 (21)	11.5 ± 3.2	11.1 ± 2.8	24 m	24 m
Tang 2010	proteinuria ≥ 1.0 g/d	MMF	Sup.	20 (6)	20 (8)	42.1 ± 2.6	43.3 ± 2.8	72 m	72 m
Ballardie 2002	sCr ≥ 130 μmol/L	CYC + AZA	Sup.	19	19	18–54 m	18–54m	24–72 m	24–72 m

Abbreviations: T treatment group, C control, S steroids, Sup. supportive care, AZA azathioprine, MMF mycophenolate mofetil, TAC tacrolimus, CYC cyclophosphamide, e-GFR estimated glomerular filtration rate, sCr creatinine, UPCR urine protein to creatinine ratio, UACR urine albumin to creatinine ratio, d day, m month, NA not available.

### The network structures

**Figure 2:**
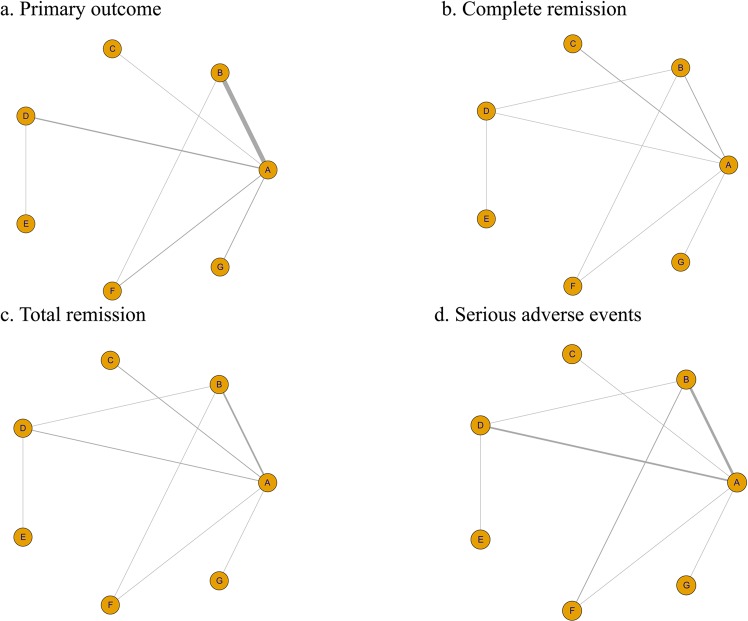
Graphic representation of comparisons of efficacy and safety for each immunosuppressive treatment for IgA nephropathy. ((**A**)Supportive care; (**B**) Steroids; (**C**) Tacrolimus; (**D**) Mycophenolate mofetil; (**E**) Cyclophosphamide; (**F**) Azathioprine, (**G**) Cyclophosphamide + Azathioprine).

Of these studies, 20 trials had provided data on primary endpoints; 12 studies had investigated secondary renal outcomes (Table 2); and 17 studies had reported serious side effects. Figure 2 exhibits the Bayesian network plot of treatment comparisons. In the diagram, the lines indicate the direct comparisons between medications. The thickness of the lines corresponds to the number of studies. The inconsistency between direct and indirect comparisons was acceptable, indicating that the model of the Bayesian approach was stable and that the results were high in reliability (Fig. S1).

**Table 2:** Definitions of primary and secondary outcomes in the selected studies.

Study	Primary Outcomes	Second Outcomes		
		Complete remission (CR)	Partial remission (PR)	Total remission (TR)
Fellstrom 2017	ESRD	NA	NA	NA
Hogg 2006	e-GFR < 60% of baseline	NA	NA	NA
Lv 2017	ESRD or death or e-GFR < 40% of baseline	NA	NA	NA
Manno 2009	doubling of baseline sCr or ESRD	NA	proteinuria < 1.0 g/d	PR
Pozzi 2004	doubling of baseline sCr	NA	NA	NA
Katafuchi 2003	ESRD	NA	NA	NA
Lv 2009	a 50% increase in sCr	NA	a 25% decrease in eGFR or 50% proteinuria reduction	PR
Harmankaya 2002	ESRD	NA	NA	NA
Lai 1986	ESRD	remission of proteinuria	NA	CR
Shoji 2000	ESRD	NA	NA	NA
Julian 1993	ESRD	NA	NA	NA
Liu 2014	a 50% increase in sCr	proteinuria < 0.4 g/d	50% proteinuria reduction	CR + PR
Hou 2017	NA	undetectable proteinuria	proteinuria 0.4–1.0 g/d	CR + PR
Hogg 2015	NA	UPCR < 0.3 g/g	50% proteinuria reduction	CR + PR
Yu 2017	a 50% increase in sCr or ESRD	UPCR < 0.2 g/g	NA	CR
Kim 2013	NA	UPCR < 0.2 g/g	50% proteinuria reduction	CR + PR
Frisch 2005	a 50% increase in sCr	NA	50% proteinuria reduction	PR
Maes 2004	a 50% increase in sCr	NA	NA	NA
Rauen 2018	ESRD	UPCR < 0.2 g/g	NA	CR
Kamei 2011	ESRD	UPCR < 0.2 g/g	UPCR 0.2–1.0 g/g	CR + PR
Pozzi 2010	a 50% increase in sCr	NA	NA	NA
Yoshikawa 2006	NA	proteinuria < 0.1 g/d	NA	CR
Tang 2010	ESRD	NA	NA	NA
Ballardie 2002	ESRD	NA	NA	NA

Abbreviations: e-GFR estimated glomerular filtration rate, sCr creatinine, UPCR urine protein to creatinine ratio, d day, NA not available.

### Primary outcomes

The number of patients who reached the primary endpoints was inversely related to the efficacy of the regimens, indicating that the higher RRs were, the worse the effect. Compared with supportive care, steroid monotherapy could significantly prevent deteriorating renal function in patients (RR = 0.28, 95% CI = 0.13–0.51). However, other immunosuppressants did not appear to be of benefit to the patients (Table 3).

**Table 3:** Network estimated Relative Risksrelative risks (RRs) of immunosuppressants on primary outcomes.

**Supportive Care**						
**0.28 (0.13, 0.51)**	**Steroids**					
1.1 (0.15, 8.4)	4.0 (0.50, 36)	**Tacrolimus**				
0.72 (0.23, 2.6)	2.6 (0.73, 12)	0.65 (0.068, 7.3)	**Mycophenolate mofetil**			
2.1 (0.23, 26.0)	7.7 (0.80, 110.0)	1.9 (0.098, 49.0)	2.9 (0.43, 25)	**Cyclophosphamide**		
0.35 (0.095, 1.2)	1.2 (0.37, 4.7)	0.31 (0.029, 3.4)	0.48 (0.077, 2.6)	0.16 (0.01, 2.1)	**Azathioprine**	
0.57 (0.17, 2.5)	2.0 (0.54, 11)	0.51 (0.052, 6.5)	0.78 (0.14, 5.0)	0.27 (0.02, 3.9)	1.6 (0.29, 12.0)	**Cyclophosphamide + Azathioprine**

Values are presented as RRs with 95% confidence intervals (CIs). The regimen listed in each row is compared with the regimen listed in each column, and RRs of <1 favor row-defining treatment.

**Figure 3:**
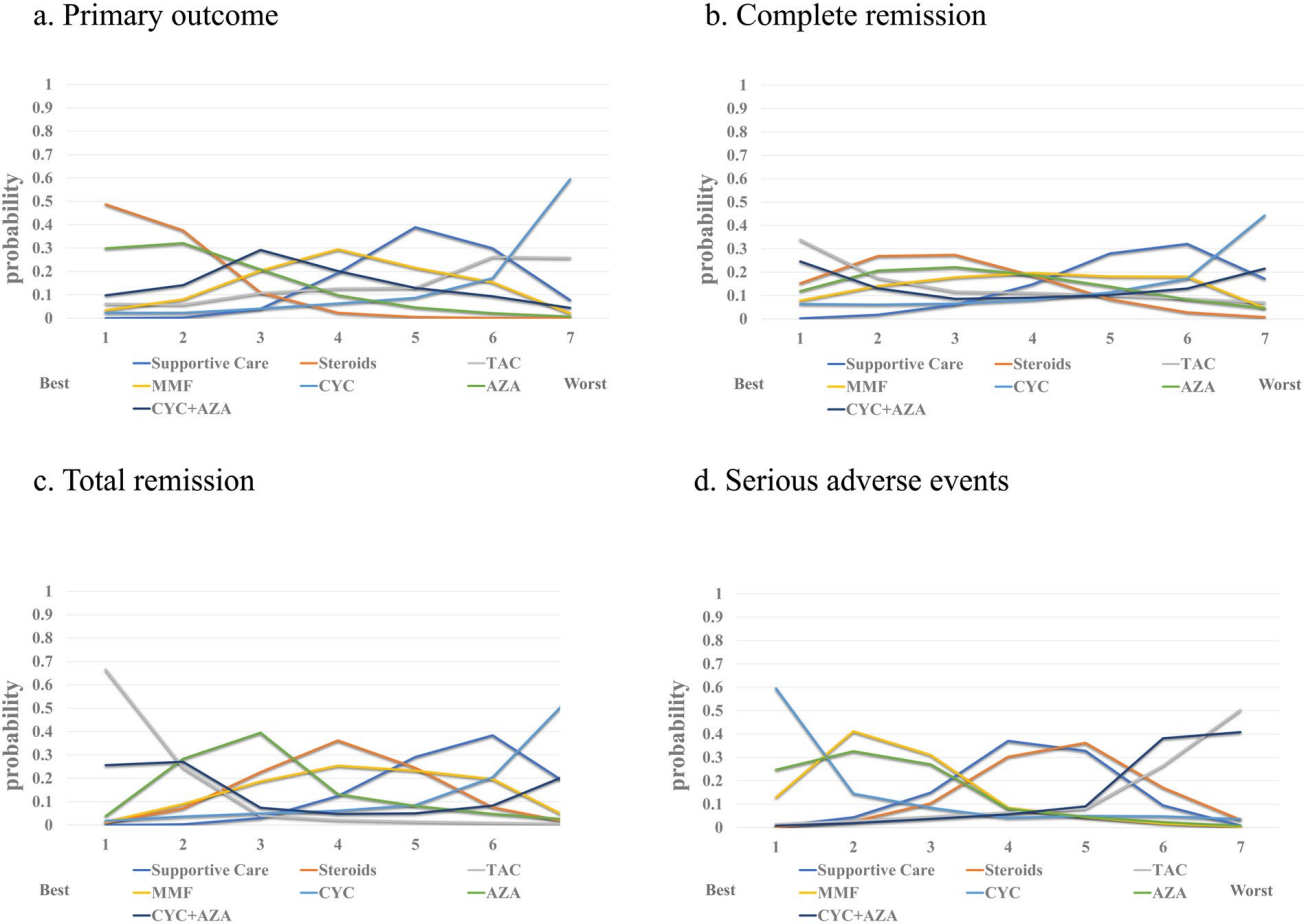
Rankings of efficacy and safety for each immunosuppressive treatment for IgA nephropathy. The numbers on the x-axis represent the priority level of the recommendation. The values on the y-axis indicate the SUCRA. For example, there was a 48.7% probability that steroids were the best choice to protect patients from ESRD and should be used as the first-line therapy. However, TAC ranked last in terms of clinical recommendation. (TAC, Tacrolimus; MMF, Mycophenolate mofetil; CYC. Cyclophosphamide; AZA, Azathioprine).

Based on the SUCRA analysis, there was a 48.7% probability that steroids were the best choice for protecting patients from ESRD (Fig. 3a). AZA was the second choice, followed by CYC + AZA. Conversely, there was a 59.4% probability that CYC was the worst option. TAC and supportive care were the penultimate options.

The heterogeneity of the comparisons of steroids versus supportive care was low (I2 = 0.0%, Fig. S2a). However, assessing the heterogeneity of other treatments indicated an I2 score = 84.5% for CYC + AZA versus supportive care, and an I2 score = 64.2% for MMF versus supportive care. The assessment of heterogeneity in the study demonstrated that the renoprotective effectiveness of steroids was robust.

### Secondary outcomes

There was no marked difference in the rates of complete remission (Table 4). However, compared with supportive care, TAC (RR = 3.1, 95% CI = 1.2–9.4) and steroids (RR = 1.2, 95% CI = 0.88–2.4) were more likely to have to provide total remission (Table 5).

**Table 4:** Network estimated Relative Risksrelative risks (RRs) of immunosuppressants on complete remission.

**Supportive Care**						
2.1 (0.59, 11.0)	**Steroids**					
2.4 (0.32, 22.0)	1.1 (0.08, 14.0)	**Tacrolimus**				
1.5 (0.17, 10.0)	0.73 (0.08, 3.5)	0.62 (0.03, 13.0)	**Mycophenolate mofetil**			
0.79 (0.03, 16.0)	0.38 (0.013, 5.8)	0.33 (0.0063, 12.0)	0.53 (0.049, 5.6)	**Cyclophosphamide**		
1.8 (0.33, 12.0)	0.88 (0.13, 4.4)	0.76 (0.047, 12.0)	1.2 (0.12, 17.0)	2.2 (0.093, 84.0)	**Azathioprine**	
1.6 (0.093, 28.0)	0.73 (0.026, 16.0)	0.65 (0.018, 22.0)	1.0 (0.035, 41.0)	2.0 (0.032, 150.0)	0.87 (0.03, 24.0)	**Cyclophosphamide + Azathioprine**

Values are presented as RRs with 95% confidence intervals (CIs). The regimen listed in each row is compared with the regimen listed in each column, and RRs of >1 favor row-defining treatment.

**Table 5:** Network estimated Relative Risksrelative risks (RRs) of immunosuppressants on total remission.

**Supportive Care**						
1.2 (0.88, 2.4)	**Steroids**					
**3.1 (1.2, 9.4)**	2.5 (0.74 7.7)	**Tacrolimus**				
1.1 (0.61, 2.6)	0.97 (0.42, 1.7)	0.38 (0.10, 1.30)	**Mycophenolate mofetil**			
0.82 (0.28, 2.9)	0.69 (0.19, 2.0)	0.27 (0.057, 1.30)	0.71 (0.29, 1.8)	**Cyclophosphamide**		
1.4 (0.79, 3.2)	1.2 (0.54, 2.1)	0.46 (0.13, 1.60)	1.2 (0.50, 3.2)	1.7 (0.46, 6.5)	**Azathioprine**	
1.7 (0.20, 13.0)	1.3 (0.14, 11.0)	0.53 (0.055, 5.1)	1.4 (0.14, 12.0)	2.0 (0.17, 20.0)	1.2 (0.12, 9.8)	**Cyclophosphamide + Azathioprine**

Values are presented as RRs with 95% confidence intervals (CIs). The regimen listed in each row is compared with the regimen listed in each column, and RRs of >1 favor row-defining treatment.

SUCRA analysis of complete remission demonstrated that TAC was the optimal choice with 33.9% probability (Fig. 3b), followed by steroids. Notably, CYC and supportive care were the two least effective strategies (SUCRA of 44.3% and 32.0%, respectively).

SUCRA analysis of total remission illustrated that TAC was the most favorable (SUCRA of 66.5%). AZA and CYC + AZA were next (SUCRA of 28.3% and 27.1%, respectively). All regimens except CYC seemed to be better than supportive care.

### Serious adverse events

The rates of severe side effects did not show any significant differences (Table 6), but TAC and CYC + AZA were the worst protocols proved by the SUCRA  analysis (Fig. 3d).

**Table 6:** Network estimated Relative Risksrelative risks (RRs) of immunosuppressants on serious side effects.

**Supportive Care**						
1.1 (0.38, 2.4)	**Steroids**					
2.9 (0.32, 38)	2.6 (0.27, 2.6)	**Tacrolimus**				
0.45 (0.088, 1.6)	0.41 (0.088, 1.6)	0.15 (0.007, 1.80)	**Mycophenolate mofetil**			
0.19 (0.003, 4.7)	0.17 (0.003, 4.7)	0.061 (0.0004, 3.0)	0.44 (0.009, 8.4)	**Cyclophosphamide**		
0.41 (0.062, 1.9)	0.38 (0.08, 1.5)	0.14 (0.006, 1.90)	0.92 (0.12, 7.0)	2.2 (0.058, 150.0)	**Azathioprine**	
2.5 (0.42, 16.0)	2.2 (0.35, 20.0)	0.87 (0.038, 15.0)	5.6 (0.70, 71.0)	14.0 (0.36, 1200.0)	6.1 (0.64, 91.0)	**Cyclophosphamide + Azathioprine**

Values are presented as RRs with 95% confidence intervals (CIs). The regimen listed in each row is compared with the regimen listed in each column, and RRs of <1 favor row-defining treatment.

### Transitivity assumption and sensitivity analysis

It should be noted that several comparisons had only a few studies, and it is difficult to statistically assess transitivity properly due to the lack of data. Generally, the transitivity was reasonable in this study due to the accurate diagnosis of IgAN. However, three important variables (age, duration of follow-up and severity) could not be neglected, as they might have influenced prognosis. Subgroup analyses were conducted to address these problems.

**Figure 4:**
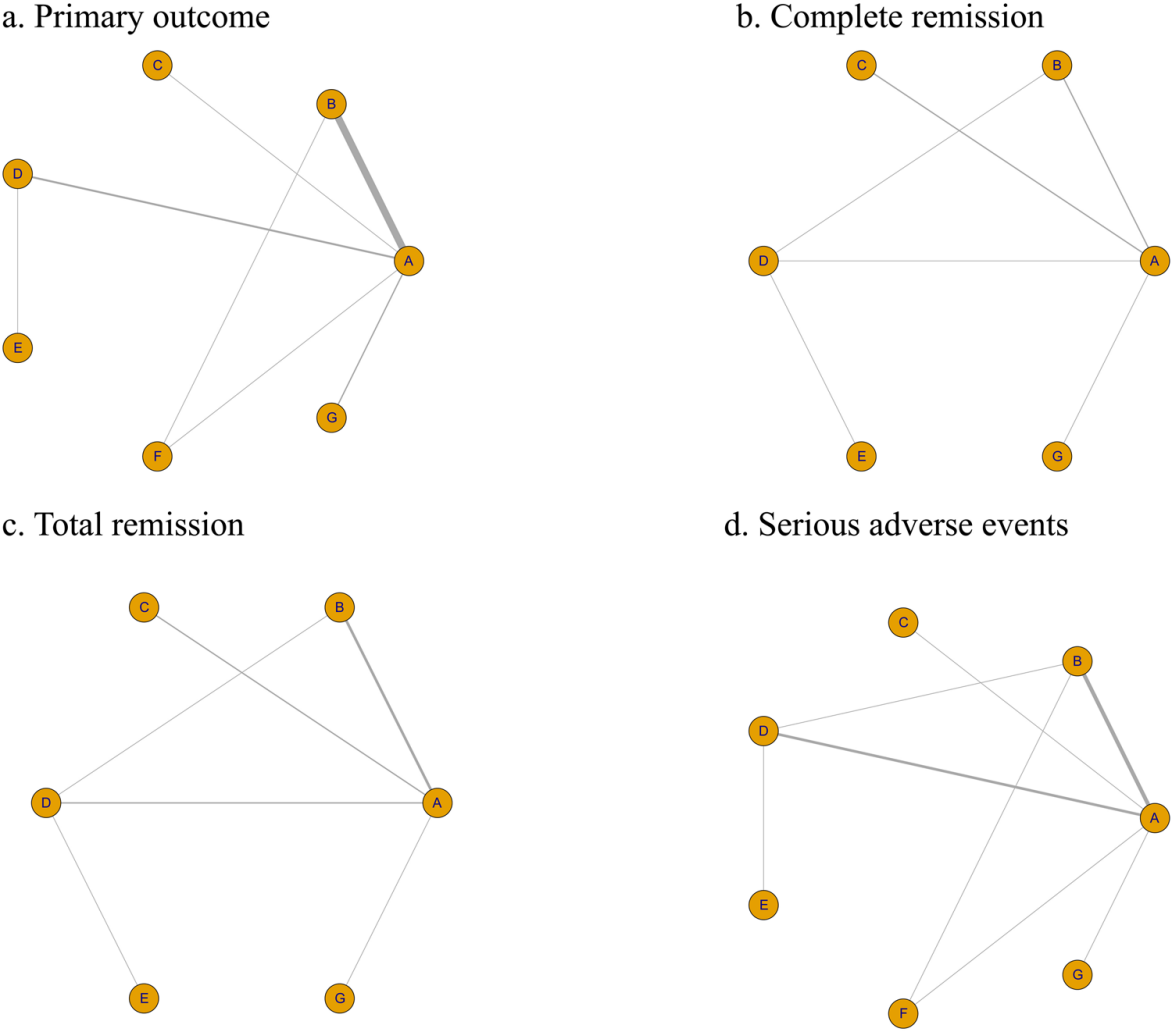
Graphic representation of comparisons of efficacy and safety for each immunosuppressive treatment for adult patients with IgA nephropathy. ((**A**) Supportive care; (**B**) Steroids; (**C**) Tacrolimus; (**D**) Mycophenolate mofetil; (**E**) Cyclophosphamide; (**F**) Azathioprine; (**G**) Cyclophosphamide + Azathioprine).

**Figure 5:**
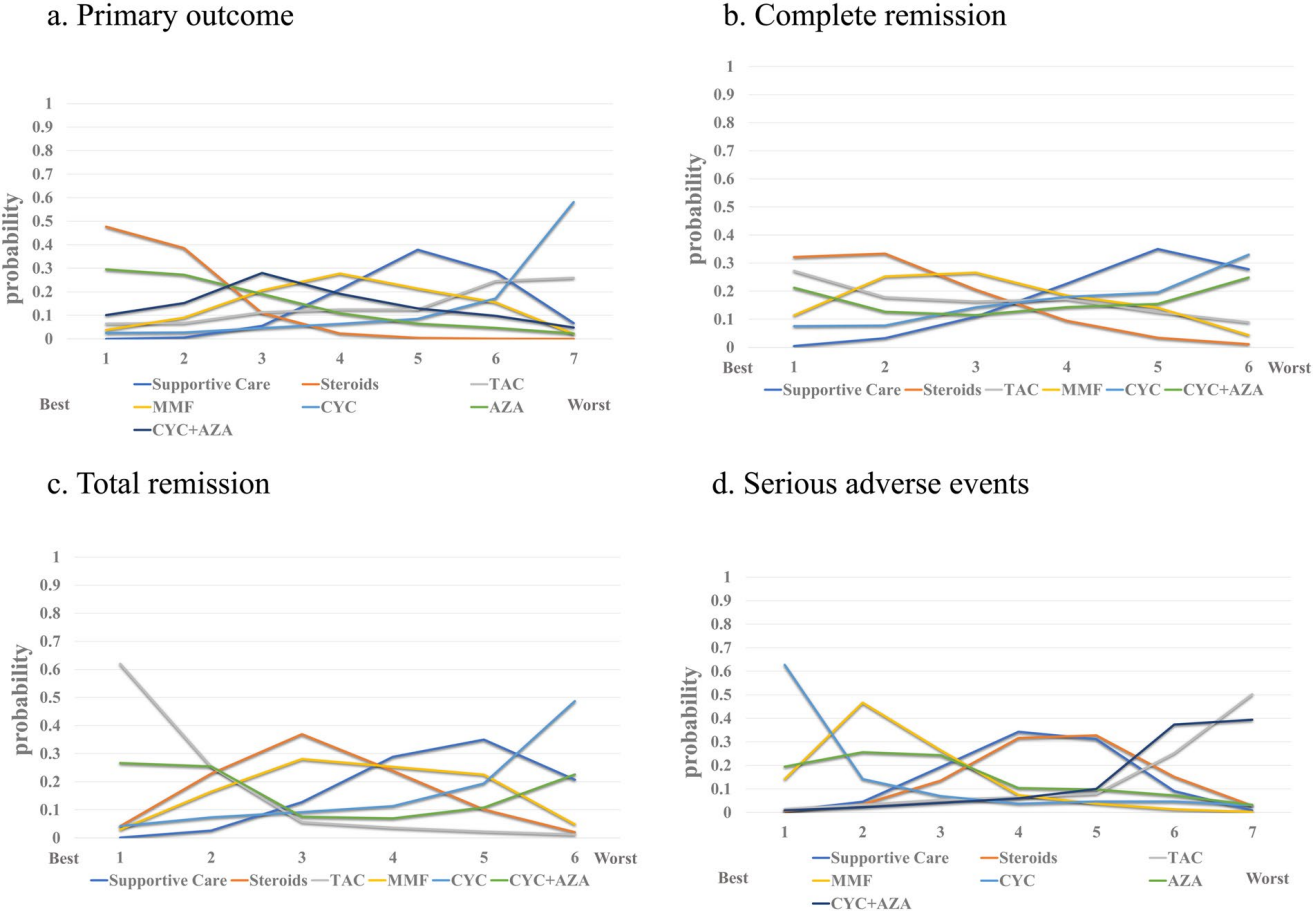
Rankings of efficacy and safety for each immunosuppressive treatment for adult patients with IgA nephropathy. The numbers on the x-axis represent the priority level of the recommendation. The values on the y-axis indicate the SUCRA. TAC, Tacrolimus; MMF, Mycophenolate mofetil; CYC. Cyclophosphamide; AZA, Azathioprine).

Some included studies investigated the efficacy of immunosuppressive therapies in the treatment of pediatric patients^26,29^. These studies were removed for the purpose of eliminating the potential effects of age. The network structures are shown in Fig. 4. In keeping with the previous results, steroid therapy had unique superiority in protecting kidney function (RR = 0.28, 95% CI = 0.12–0.54, Fig. S3). In addition, SUCRA analysis of complete remission also indicated that steroids were the best regimen (SUCRA of 47.6%, Fig. 5a). Accordingly, steroids might be recommended as the first-line therapy . Adult patients treated with TAC seemed to have more total remissions than patients treated with other drugs (RR = 3.1, 95% CI = 0.91–12.0, SUCRA of 62.0%, Figs. S3 and 5c), but TAC seemed to have no positive impact on the primary outcome and had relatively high rates of serious side effects (Fig. 5a,d). Notably, the serious side effects of CYC + AZA and TAC tended to be more obvious than those of the other drugs, although there was no significant difference.

Subgroup analysis of adult patients with proteinuria >0.75 g/d was carried out to reduce heterogeneity^8–14,18–24,28^. Notably, no patients received TAC treatment in this subgroup. A similar conclusion was reached: steroids could significantly prevent patients from deteriorating renal function and might be recommended first (Fig. S4). CYC + AZA might have advantages in total remission but had no effects on primary outcomes.

Considering that different follow-up times might lead to different outcomes, the adult patients with a follow-up period >24 months were reanalyzed^8,9,11–15,18,20,22,25,27,28,30,32^. It should be noted that indirect comparisons of complete and total remissions could not be conducted because the key variables were lacking (Fig. S5). However, the analysis of primary outcomes and serious adverse events similarly illustrated that steroids were the best treatment for reducing the number of patients progressing to ESRD.

## Discussion

IgAN is the most common primary glomerulonephritis worldwide and can gradually progress to ESRD [3]. Patients with ESRD suffer and under tremendous economic pressure. For these patients, prevention of ESRD makes a large difference. As IgAN is an immune-mediated disease, immunosuppressive therapies may provide clinical recovery or remission, especially for patients who are not responsive to available regimens effectively^4^. A previous meta-analysis indicated that immunosuppressants were a promising regimen to treat IgAN because they have a significant ability to reduce the level of urine protein with acceptable side effects^5^. However, their ability to protect renal function was not investigated, which was much more important. Recently, some scholars have proposed the STOP-IgAN plan, which suggests that immunosuppression cannot protect patients from progression according to a high-quality randomized controlled trial^8,33^. Therefore, the options for immunosuppressive agents are intensely debated. To find the first-line immunosuppressive treatments of IgAN, this network meta-analysis evaluated the efficacy and safety of different immunosuppressive agents for patients with IgAN.

Our network meta-analysis found that steroids might be the most effective immunosuppressive therapy among all immunosuppressants to prevent ESRD in IgAN patients. The forest plot of RRs demonstrated that steroid monotherapy was the immunosuppressive therapy that could significantly reduce the number of patients suffering from deterioration of renal function, compared with supportive care. SUCRA analysis of primary outcomes also supported the view that steroid monotherapy was the preferred immunosuppressive strategy. Meanwhile, there was a relatively obvious trend of steroids having an advantage in terms of complete remissions and total remissions although no statistical significance was found. The rates of serious adverse events were also acceptable because there was no significant difference compared with supportive care. The heterogeneity was also low. Therefore, it can be concluded that steroids should be the first-line immunosuppressive therapy. Similarly, AZA might be a second-best strategy for treating IgAN. The conclusion of this network meta-analysis was consistent with that of recent RCTs, which indicated that oral methylprednisolone and targeted-release budesonide could have potential renal benefits^9,10^. However, the STOP-IgAN trial has demonstrated that administration of immunosuppressants, including steroids can decrease proteinuria levels transiently but cannot prevent decreases in the e-GFR^8,33^. Notably, in the STOP-IgAN cohort, 1 out of 55 patients in the steroid monotherapy group and 5 out of 54 patients in supportive care group progressed to ESRD, demonstrating a slight trend for steroids being superior to supportive care, although there was no statistical significance, which might have resulted from the relatively small number of patients. Moreover, some retrospective studies have reported that immunosuppression is effective for treating IgAN^34,35^. Therefore, we believe that the addition of steroids to supportive care is beneficial to patients with IgAN.

TAC might be superior in terms of inducing a reduction in urine protein. However, TAC was the least effective at preventing the progression of IgAN, leading to more patients suffering from ESRD as well as intolerable side effects. A previous pairwise meta-analysis clearly concluded that TAC was a promising drug for IgAN without an increased risk of side effects, but these results were not exactly the same as ours^36^. That previous study did not take renal outcomes into consideration. Meanwhile, severe adverse events and mild side effects were not differentiated. Notwithstanding the fact that both that study and our study proposed the distinct efficacy of proteinuria reduction in the short term, our study also indicated that TAC might not improve renal function significantly during long-term follow-up and might not be superior to supportive care. In addition, a higher risk of side effects, including cardiovascular, gastrointestinal, genitourinary, neurological, hematologic and other symptoms, suggests that TAC should not be recommended for IgAN.

Previous studies on the efficacy of MMF and CYC + AZA have been contradictory. A comprehensive analysis of existing articles showed that both MMF and CYC did not demonstrably lower the risks for renal deterioration. To interpret appropriately, the results need context. Genetic factors are one of the most important causes of IgAN. Renal survival and reactions to drugs are dramatically different among populations^37^. Our network meta-analysis found that Chinese patients were sensitive to MMF, while patients from America showed a poor response to MMF therapy^19–21,24^. Race was probably the primary source of this heterogeneity. Different clinical manifestations, ages, and histopathological characteristics of the included patients, the therapeutic method and the duration of follow-up might also have contributed to the heterogeneity. Due to the limited number of original articles and the lack of direct comparisons, it was difficult to carry out the subgroup analysis, and the efficacies of MMF and CYC + AZA are still unclear. Further high-quality studies are required.

There were some potential limitations in our network meta-analysis. First, since different studies had their own outcome indicators, the primary and secondary outcomes were heterogeneous. Second, the histopathology of the lesions was also not assessed, and different morphologies might respond to steroids differently. Third, the dosage of immunosuppressants and period of curative time varied in different studies. The usage of steroids was also not uniform because we did not separate prednisone, methylprednisolone and budesonide. Fourth, heterogeneity did exist in the studies, but a practical method to eliminate it is lacking owe to the limited data. Consequently, multicenter RCTs with sufficient data are required to confirm our results.

### Conclusion

According to SUCRA analysis with a Bayesian approach, steroids are the best choice for the treatment of IgAN. AZA combined with steroids might be an alternative immunosuppressive therapy. TAC might decrease proteinuria but cannot improve renal function, and its side effects cannot be neglected. CYC showed no superiority in the treatment of IgAN. In summary, our study demonstrated that steroids should be recommended as the first-line immunosuppressive therapy for IgAN.

## Supplementary information

Supplementary information.
