# Supplementary material for: The efficacy and safety of immunosuppressive therapies in the treatment of IgA nephropathy: A network meta-analysis
Source: Sci Rep. 2020 Apr 8;10:6062. doi: 10.1038/s41598-020-63170-w (PMC7142138; doi:10.1038/s41598-020-63170-w)
Supplement: Supplementary file 1 — Supplementary information. [file 41598_2020_63170_MOESM1_ESM.pdf]

The efficacy and safety of immunosuppressive therapies in the treatment of IgA nephropathy: A network meta-analysis

Jiaxing Tan, Lingqiu Dong, Donghui Ye, Yi Tang, Tengyue Hu, Zhengxia Zhong, Padamata Tarun, Yicong Xu, Wei Qin

Fig. S1. Inconsistency using the node-splitting approach.

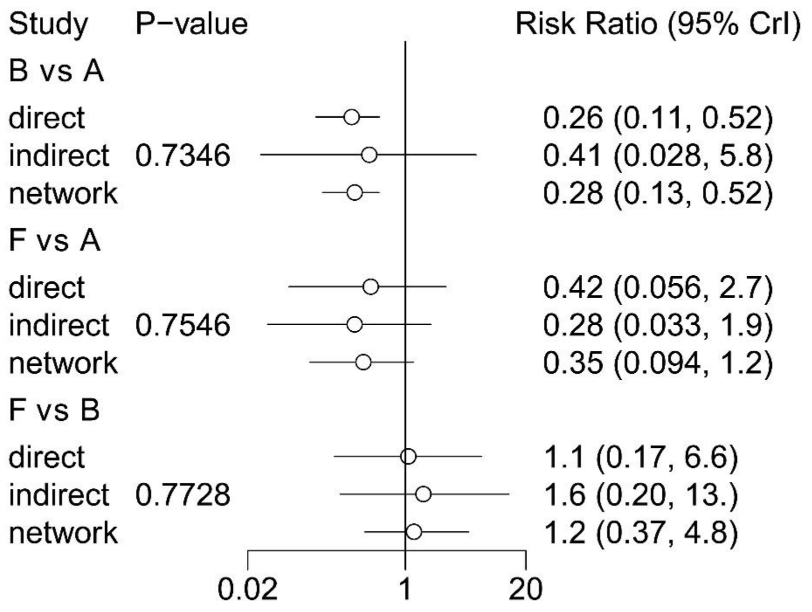

Fig. S1a. Inconsistency using the node-splitting approach for primary outcomes. (A, Supportive care; B, Steroids; C, Tacrolimus; D, Mycophenolate mofetil; E, Cyclophosphamide; F, Azathioprine; G, Cyclophosphamide+ Azathioprine)

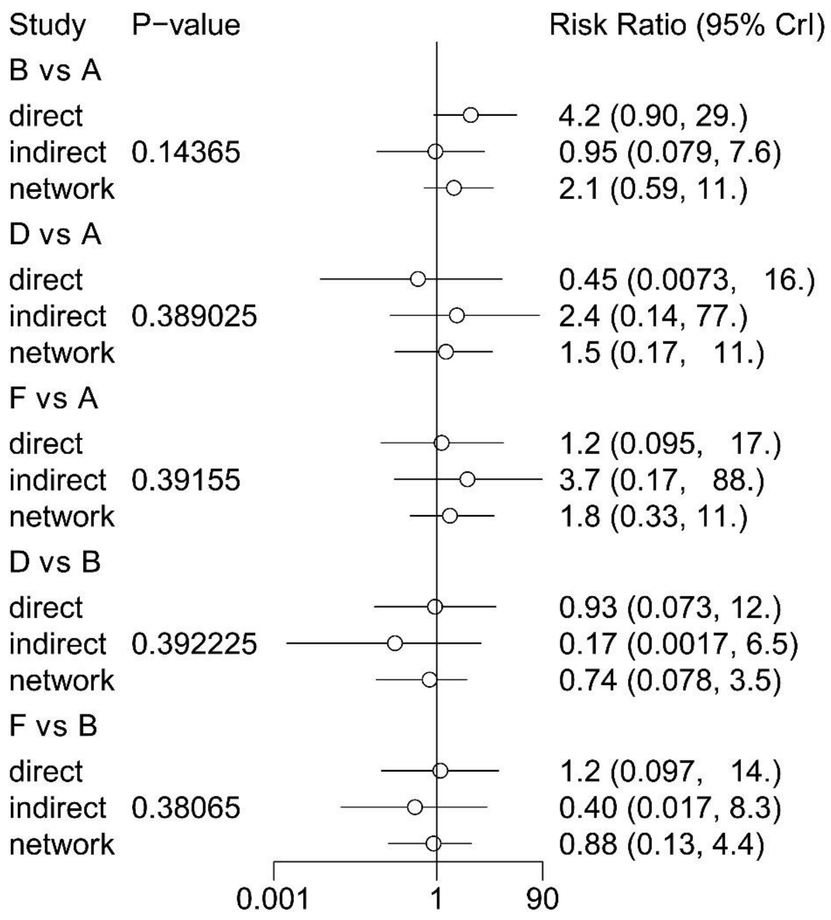

Fig. S1b. Inconsistency using the node-splitting approach for complete remission. (A, Supportive care; B, Steroids; C, Tacrolimus; D, Mycophenolate mofetil; E, Cyclophosphamide; F, Azathioprine; G, Cyclophosphamide+ Azathioprine)

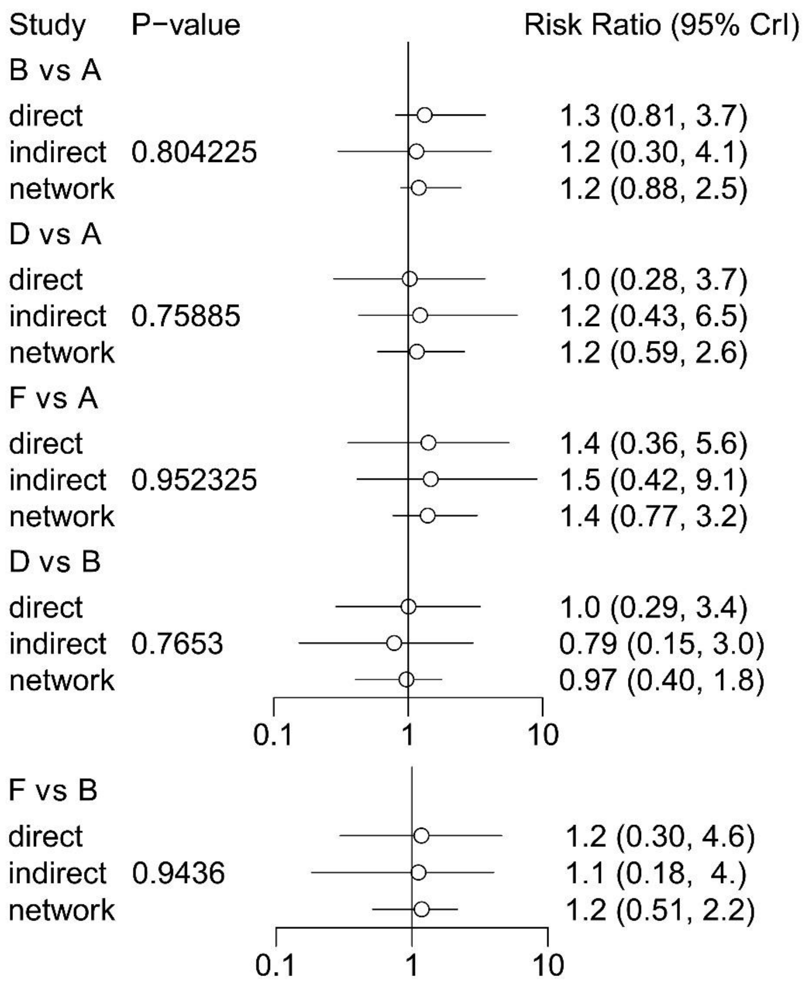

Fig. S1c. Inconsistency using the node-splitting approach for total remission. (A, Supportive care; B, Steroids; C, Tacrolimus; D, Mycophenolate mofetil; E, Cyclophosphamide; F, Azathioprine; G, Cyclophosphamide+ Azathioprine)

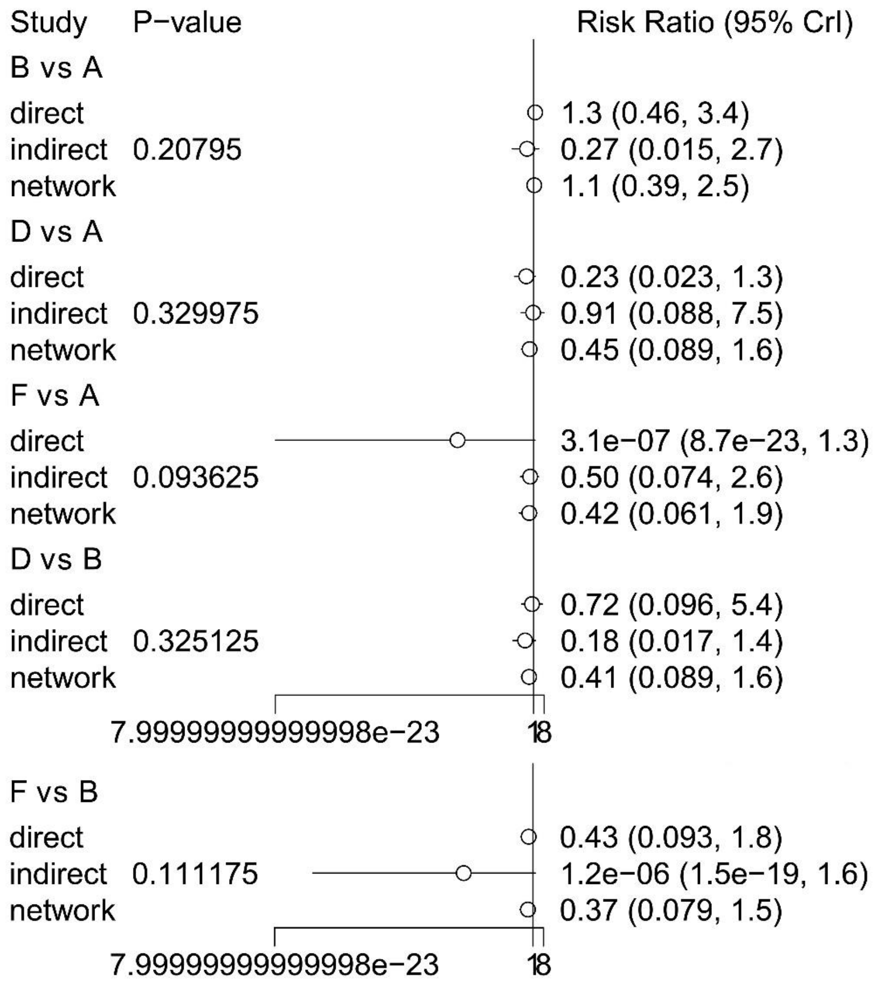

Fig. S1d. Inconsistency using the node-splitting approach for serious adverse events. (A, Supportive care; B, Steroids; C, Tacrolimus; D, Mycophenolate mofetil; E, Cyclophosphamide; F, Azathioprine; G, Cyclophosphamide+ Azathioprine)

Fig. S2. Heterogeneity analysis on endpoints.

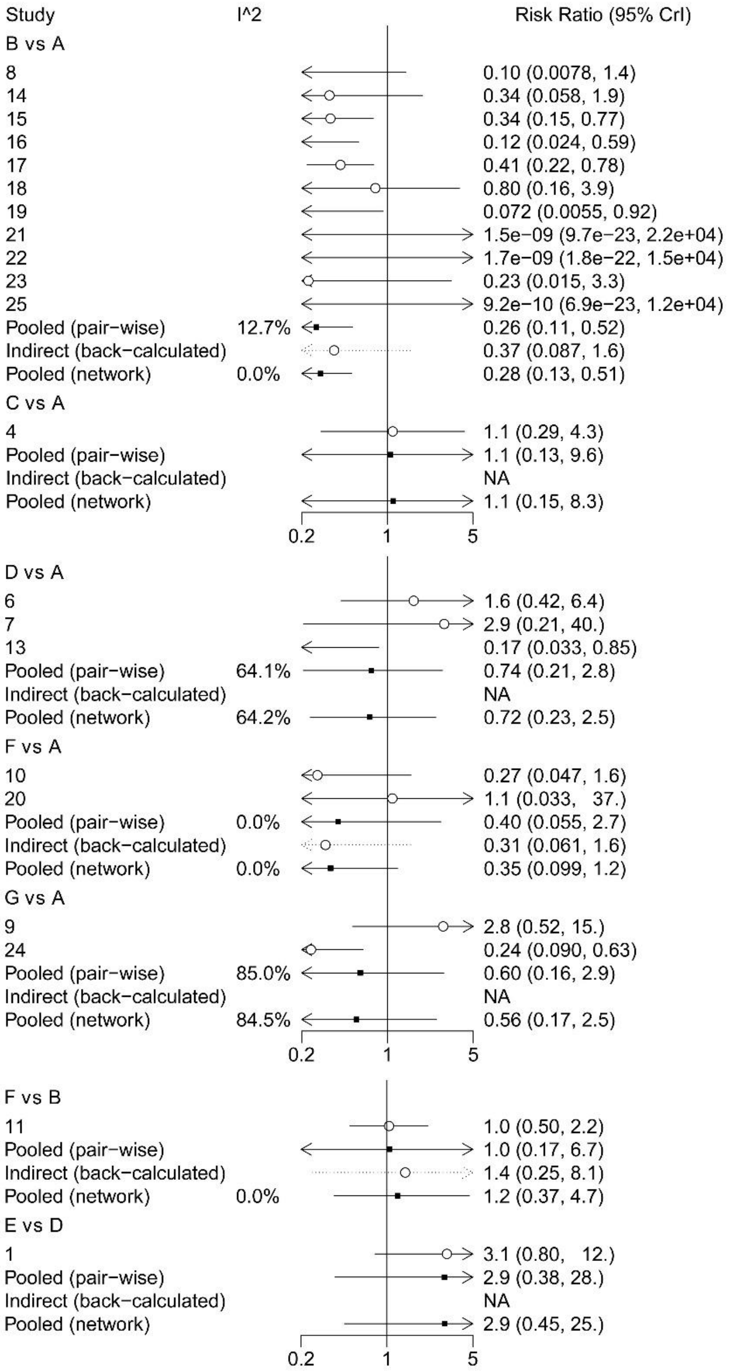

Fig. S2a. Heterogeneity analysis on primary outcomes. (A, Supportive care; B, Steroids; C, Tacrolimus; D, Mycophenolate mofetil; E, Cyclophosphamide; F, Azathioprine; G, Cyclophosphamide+ Azathioprine)

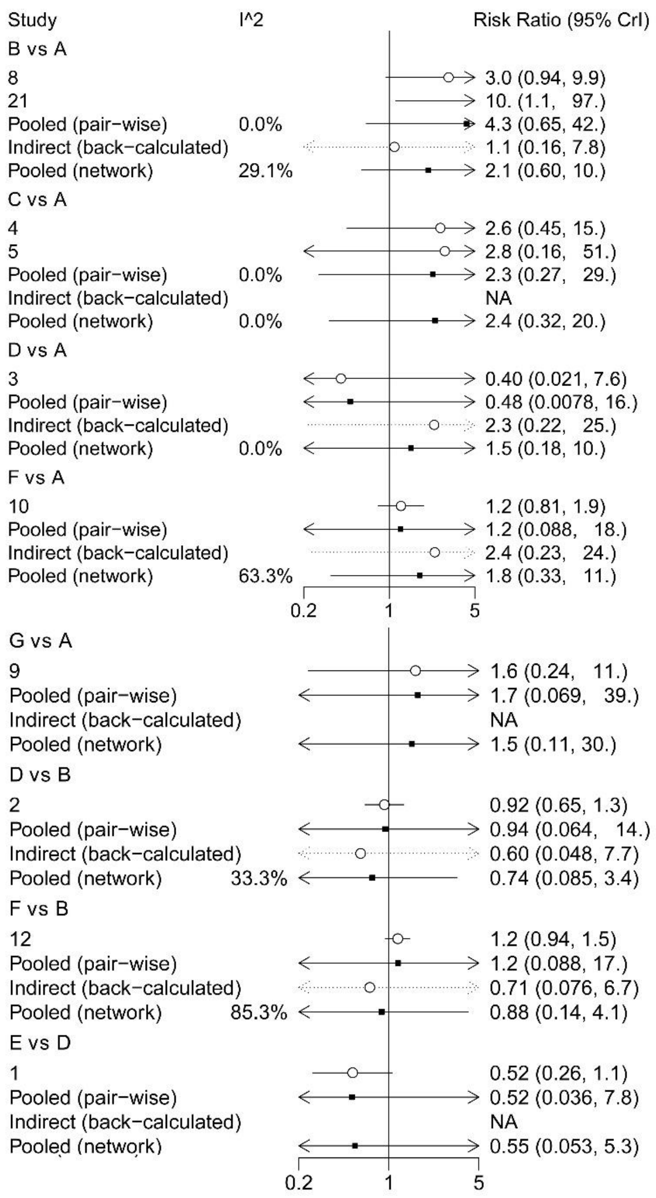

Fig. S2b. Heterogeneity analysis on complete remission. (A, Supportive care; B, Steroids; C, Tacrolimus; D, Mycophenolate mofetil; E, Cyclophosphamide; F, Azathioprine; G, Cyclophosphamide+ Azathioprine)

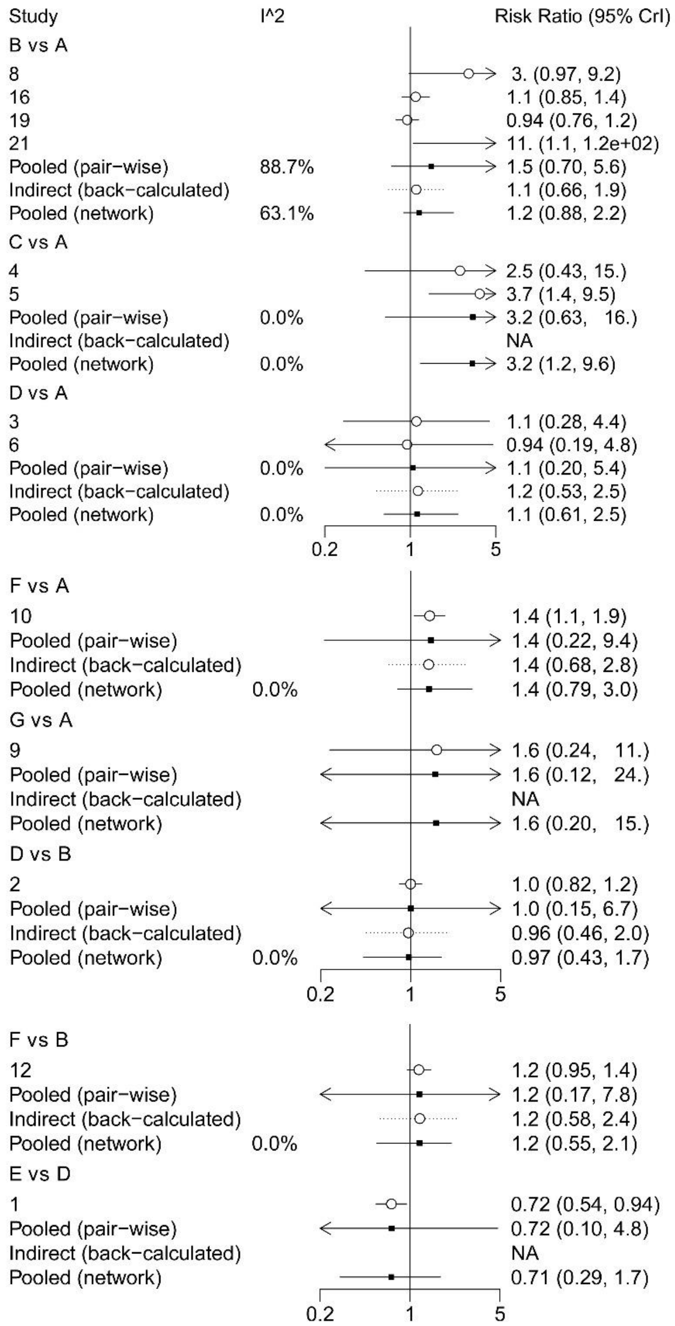

Fig. S2c. Heterogeneity analysis on total remission. (A, Supportive care; B, Steroids; C, Tacrolimus; D, Mycophenolate mofetil; E, Cyclophosphamide; F, Azathioprine; G, Cyclophosphamide+ Azathioprine)

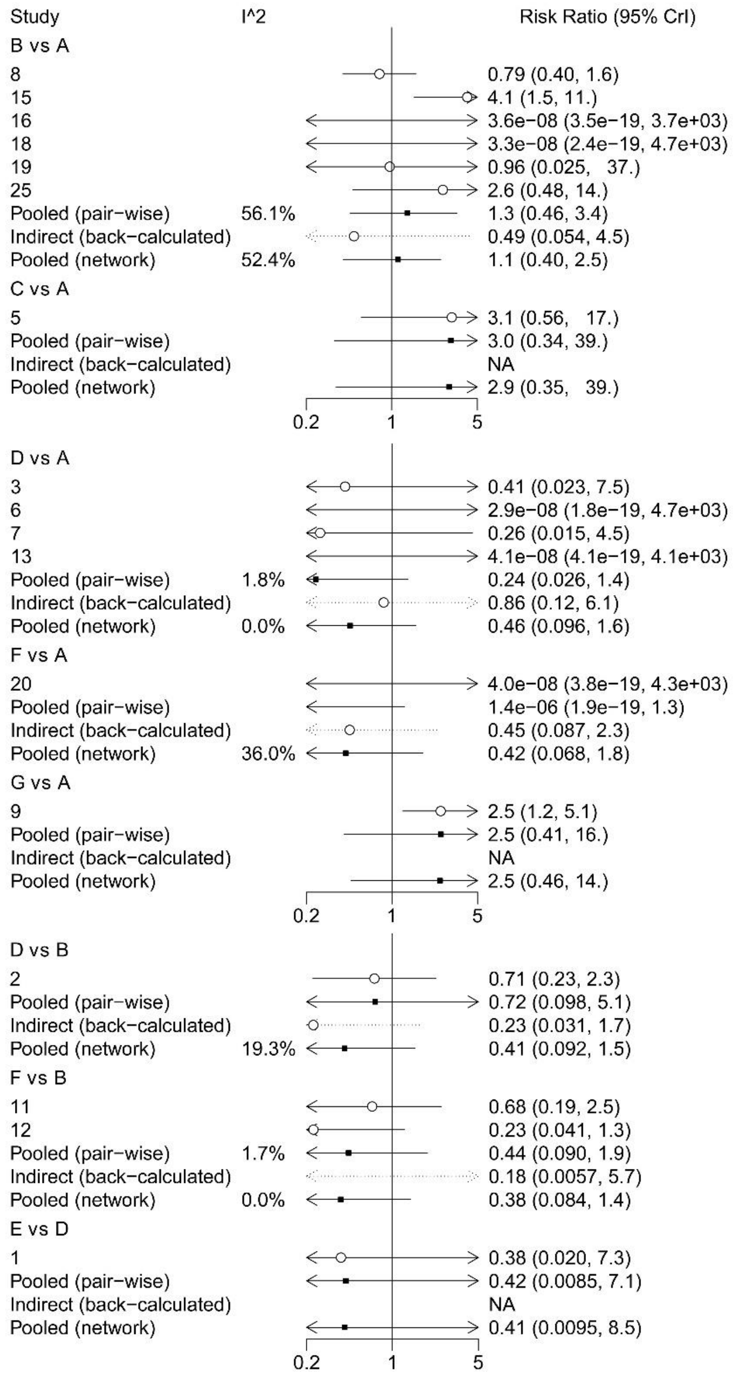

Fig. S2d. Heterogeneity analysis on serious adverse events. (A, Supportive care; B, Steroids; C, Tacrolimus; D, Mycophenolate mofetil; E, Cyclophosphamide; F, Azathioprine; G, Cyclophosphamide+ Azathioprine)

Fig. S3. Network estimated relative risks (RRs) of immunosuppressants on different outcomes in adult patients. (A, Supportive care; B, Steroids; C, Tacrolimus; D, Mycophenolate mofetil; E, Cyclophosphamide; F, Azathioprine; G, Cyclophosphamide+ Azathioprine)

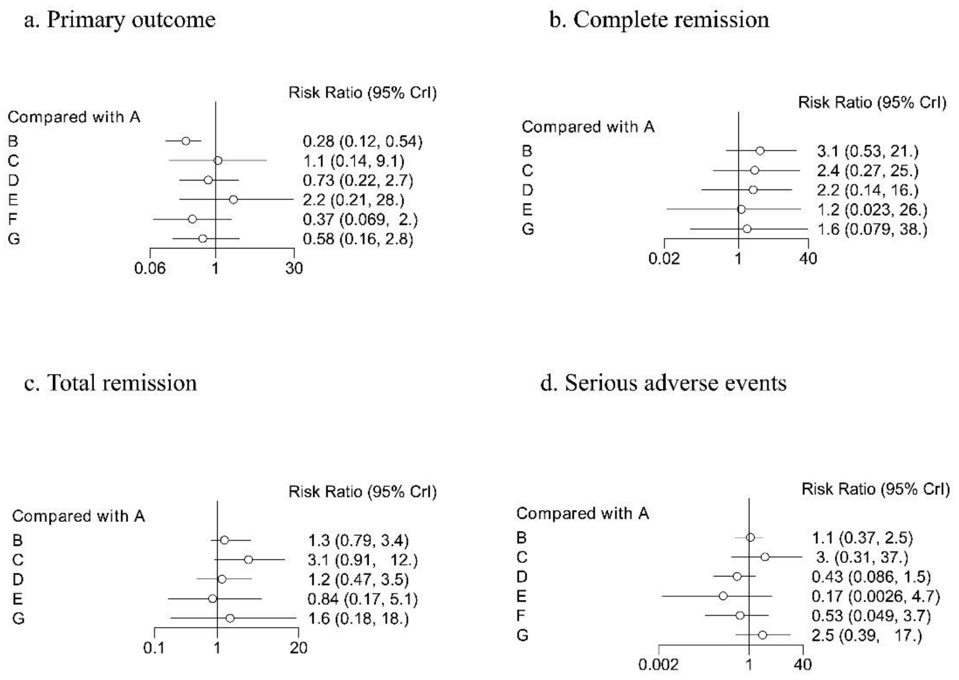

Fig. S4: The results of adult patients with proteinuria>0.75 g/24h.

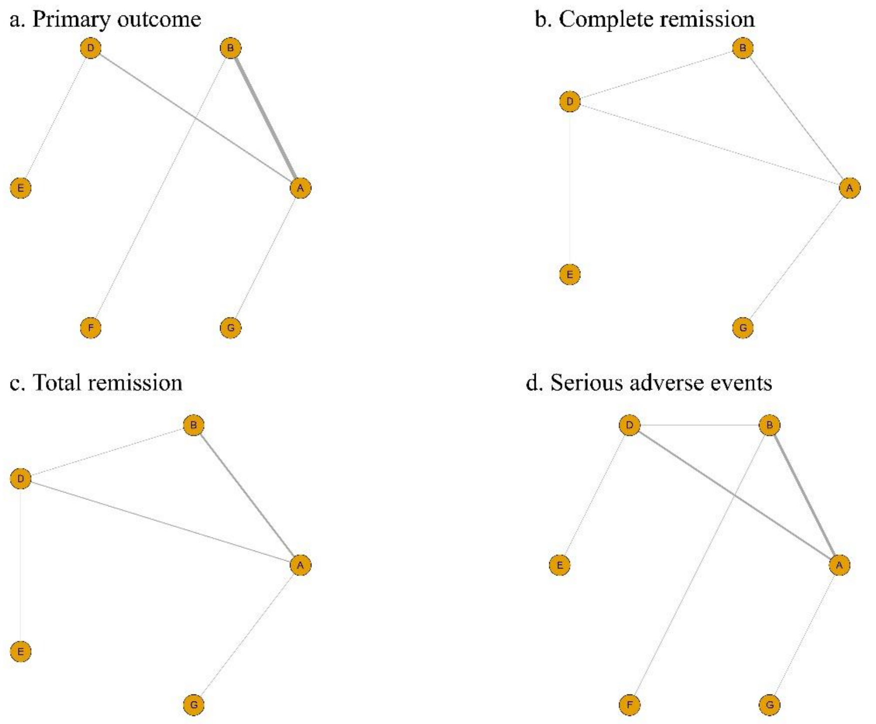

Fig. SS 4a. The network diagram of primary outcomes. (A, Supportive care; B, Steroids; C, Tacrolimus; D, Mycophenolate mofetil; E, Cyclophosphamide; F, Azathioprine; G, Cyclophosphamide+ Azathioprine)

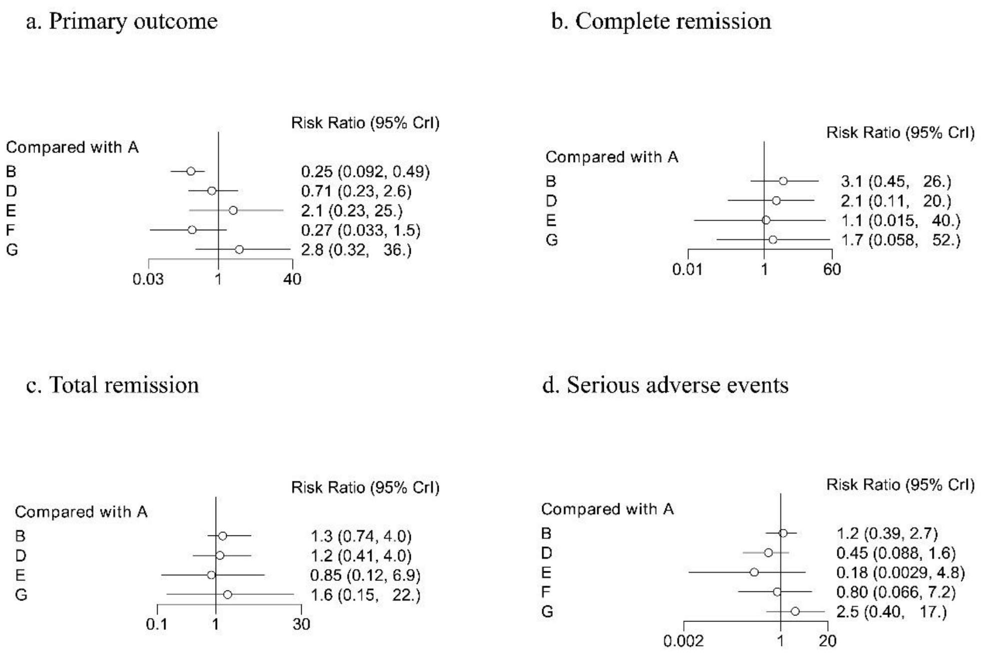

Fig. S4b. Network estimated Relative Risksrelative risks (RRs) of immunosuppressants on different outcomes. (A, Supportive care; B, Steroids; C, Tacrolimus; D, Mycophenolate mofetil; E, Cyclophosphamide; F, Azathioprine; G, Cyclophosphamide+ Azathioprine)

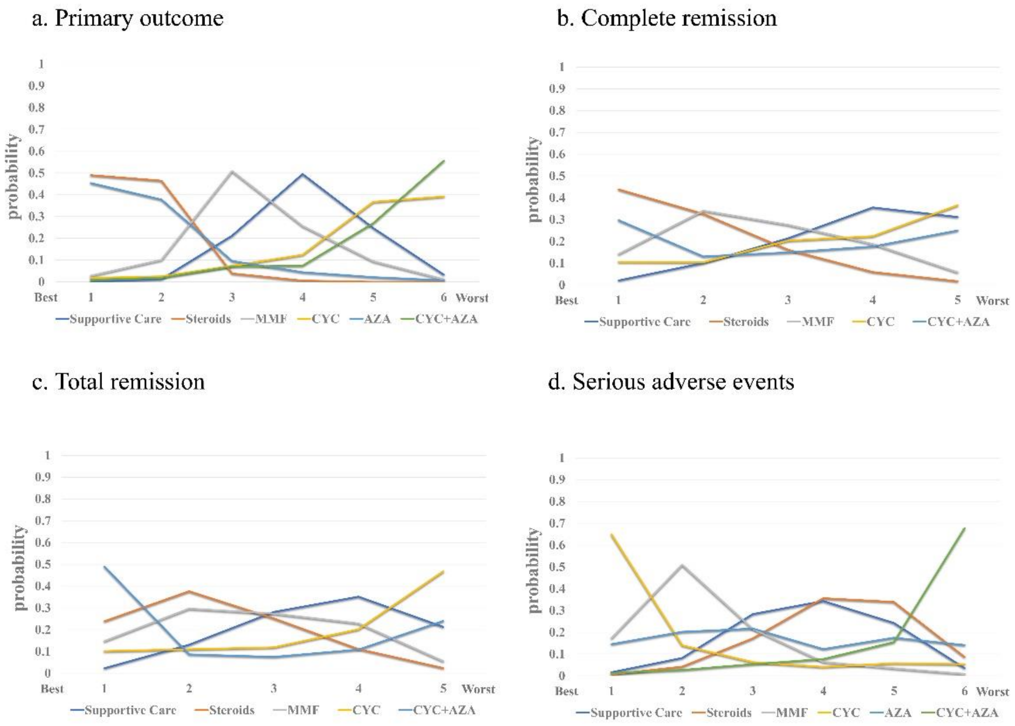

Fig. S4c. Rankings of efficacy and safety for each immunosuppressive treatment for adult patients with proteinuria>0.75 g/24h. The numbers on the x-axis represent the priority level of the recommendation. The values on the y-axis indicate the SUCRA. TAC, Tacrolimus; MMF, Mycophenolate mofetil; CYC. Cyclophosphamide; AZA, Azathioprine)

Fig. S5: The results of adult patients with follow-up period > 24 months.

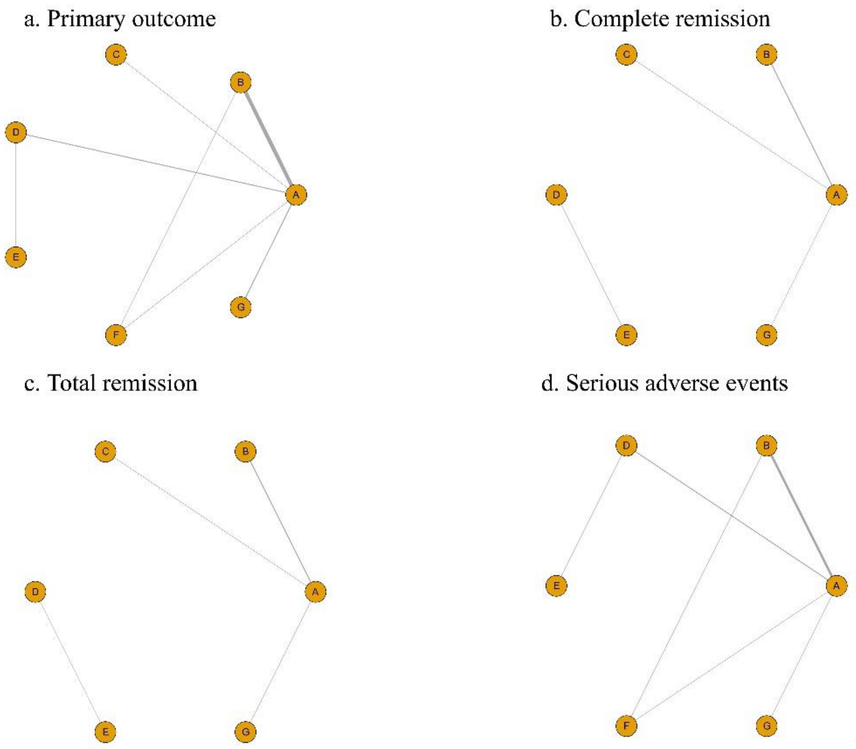

Fig. S5a. The network diagram of primary outcomes. (A, Supportive care; B, Steroids; C, Tacrolimus; D, Mycophenolate mofetil; E, Cyclophosphamide; F, Azathioprine; G, Cyclophosphamide+ Azathioprine)

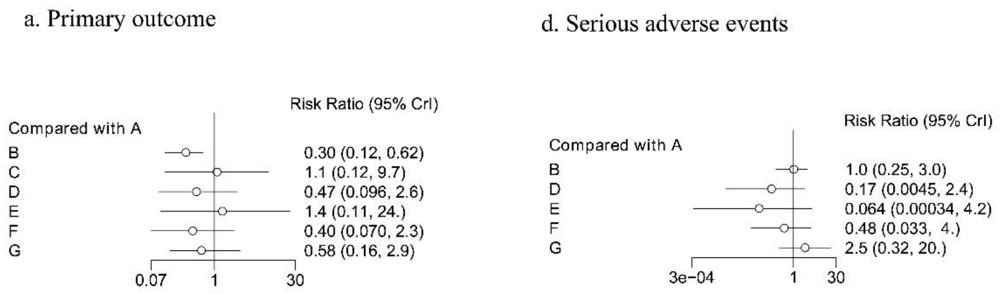

Fig. S5b. Network estimated Relative Risks (RRs) of immunosuppressants on different outcomes. (A, Supportive care; B, Steroids; C, Tacrolimus; D, Mycophenolate mofetil; E, Cyclophosphamide; F, Azathioprine; G, Cyclophosphamide+ Azathioprine)

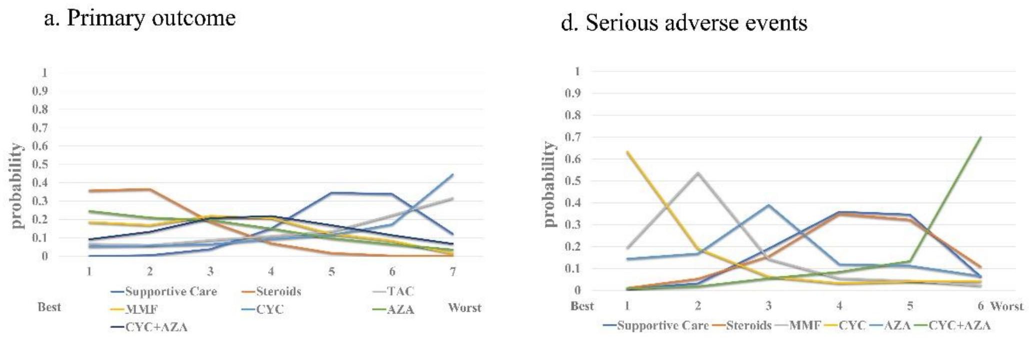

Fig. S5c. Rankings of efficacy and safety for each immunosuppressive treatment for adult patients with follow-up period > 24 months. The numbers on the x-axis represent the priority level of the recommendation. The values on the y-axis indicate the SUCRA. TAC, Tacrolimus; MMF, Mycophenolate mofetil; CYC. Cyclophosphamide; AZA, Azathioprine)

Fig. S6: Funnel plot graphics evaluating the publication bias. (A, Supportive care; B, Steroids; C, Tacrolimus; D, Mycophenolate mofetil; E, Cyclophosphamide; F, Azathioprine; G, Cyclophosphamide+ Azathioprine)

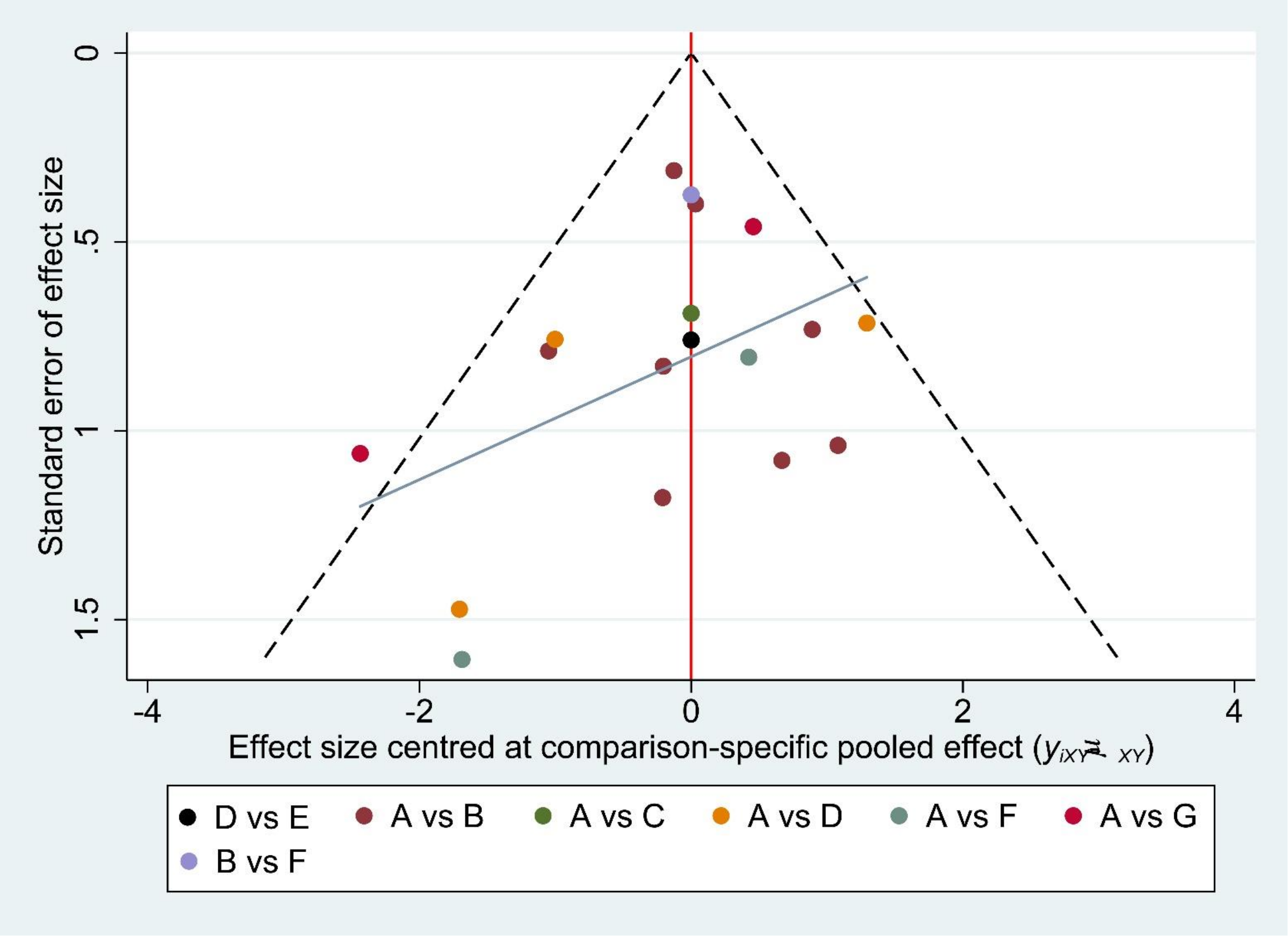

Table S1. Search strategy for the systematic review and network meta-analysis

1. MEDLINE search strategy

#1. IgA nephropathy

(((((((Glomerulonephritis, IGA[MeSH Terms]) OR Glomerulonephritis, IGA) OR IgA nephropathy) OR immunoglobulin A nephropathy) OR IgA nephritis) OR IgA glomerulonephritis) OR Berger’s disease) OR IgAN

#2. Cyclophosphamide

(((((((((((((((((((((((Cyclophosphamide[MeSH Terms]) OR Cyclophosphamide OR Cytophosphane) OR Cyclophosphamide Monohydrate) OR Monohydrate, Cyclophosphamide) OR Cyclophosphane) OR Cyclophosphamide Anhydrous) OR Anhydrous, Cyclophosphamide) OR Cyclophosphamide, (R)-Isomer) OR Cyclophosphamide, (S)-Isomer) OR Cytophosphan) OR Endoxan) OR Neosar) OR NSC-26271) OR NSC 26271) OR NSC26271) OR Procytox) OR Sendoxan) OR B-518) OR B 518) OR B518) OR Cytoxan)) OR Cyclophosphamide

#3. Azathioprine

((((((((((Azathioprine[MeSH Terms]) OR Azothioprine) OR Imurel) OR Imuran) OR Immuran) OR Azathioprine Sodium) OR Sodium, Azathioprine) OR Azathioprine Sodium Salt) OR Azathioprine Sulfate)) OR Azathioprine

#4. Tacrolimus

((((((((((((((Tacrolimus[MeSH Terms]) OR Prograf) OR Prograft) OR FR-900506) OR FR 900506) OR FR900506) OR Anhydrous Tacrolimus) OR Tacrolimus, Anhydrous) OR Tacrolimus Anhydrous) OR Anhydrous, Tacrolimus) OR FK-506) OR FK 506) OR FK506)) OR Tacrolimus

#5. Mycophenolic Acid

((((((((((((((Mycophenolic Acid[MeSH Terms]) OR Mycophenolic Acid) OR Mycophenolate Mofetil) OR Mofetil, Mycophenolate) OR Mycophenolic Acid Morpholinoethyl Ester) OR Cellcept) OR Mycophenolate Sodium) OR Sodium Mycophenolate) OR Mycophenolate, Sodium) OR Myfortic) OR Mycophenolate Mofetil Hydrochloride) OR Mofetil Hydrochloride, Mycophenolate) OR RS 61443) OR RS-61443) OR RS61443.

#6. Steroids

((Glucocorticoids[MeSH Terms]) OR Adrenal Cortex Hormones[MeSH Terms]) OR Steroids[MeSH

Terms]) OR Dexamethasone[MeSH Terms]) OR Prednisolone[MeSH Terms]) OR Prednisone[MeSH Terms]) OR Hydrocortisone[MeSH Terms]) OR Cortisone[MeSH Terms]) OR Budesonide[MeSH Terms]) OR Beclomethasone[MeSH Terms]) OR Glucocorticoid) OR Adrenal Cortex Hormones) OR Steroids) OR Dexamethasone) OR Prednisolone) OR Prednisone) OR Hydrocortisone) OR Cortisone) OR Budesonide) OR Beclomethasone) OR Glucocorticoid) OR Hormones, Adrenal Cortex) OR Corticosteroids) OR Corticoids) OR Methylfluorprednisolone) OR Hexadecadrol) OR Decameth) OR Decaspray) OR Dexasone) OR Dexpak) OR Maxidex) OR Millicorten) OR Oradexon) OR Decaject) OR Decaject-L.A.) OR Decaject L.A.) OR Hexadrol) OR Dehydrocortisone) OR delta-Cortisone) OR Rectodelt) OR Prednison Hexal) OR Sterapred) OR Ultracorten) OR Winpred) OR Apo-Prednisone) OR Cortan) OR Cortancyl) OR Panafcort) OR Cutason) OR Decortin) OR Dacortin) OR Decortisyl) OR Deltasone) OR Encortone) OR Encorton) OR Enkortolon) OR Kortancyl) OR Liquid Pred) OR Meticorten) OR Orasone) OR Panasol) OR Predni Tablinen) OR Prednidib) OR Predniment) OR Prednison Acsis) OR Acsis, Prednison) OR Pronisone) OR Sone) OR Prednison Galen) OR Predate) OR Predonine) OR Di-Adreson-F) OR Di Adreson F) OR DiAdresonF) OR Cortisone Acetate) OR 17-Hydroxy-3,11,20-trioxopregn-4-en-21-yl acetate) OR Cortone Acetate) OR Adreson) OR Budesonide, (S)-Isomer) OR Pulmicort) OR Rhinocort) OR Budesonide, (R)-Isomer) OR Horacort) OR Beclometasone) OR Asmabec Clickhaler) OR Ascocortonyl) OR Beclamet) OR Beclo Asma) OR Beclo AZU) OR Beclocort) OR Beclomet) OR Bemedrex Easyhaler) OR Beclomethasone Dipropionate) OR Dipropionate, Beclomethasone) OR Beclorhinol) OR Sanasthmax) OR Becloturmant) OR Beclovent) OR Beconase) OR Becloforte) OR Becodisk) OR Becodisks) OR Propaderm) OR Becotide) OR Sanasthmyl) OR Beconase AQ) OR Bronchocort) OR Junik) OR Qvar) OR Ecobec) OR Beclazone) OR Beclazone Easy Breathe) OR Ventolair) OR Prolair) OR Filair) OR Filair Forte) OR AeroBec Forte) OR Aerobec) OR Nasobec Aqueous) OR Respocort) OR Vancenase) OR Vanceril) OR Aldecin) OR Viarin) OR Apo-Beclomethasone

#1 AND (#2 OR #3 OR #4 OR #5 OR #6)

EMBASE search strategy

#1. IgA nephropathy

'immunoglobulin A nephropathy'/exp OR 'berger disease':ab,ti OR 'glomerulonephritis, iga':ab,ti OR 'IgA nephropathy':ab,ti OR 'Glomerulonephritis, IGA':ab,ti OR 'IgA nephritis':ab,ti OR 'IgA glomerulonephritis':ab,ti OR 'IgAN':ab,ti OR 'Bergers disease':ab,ti

#2. Cyclophosphamide

'cyclophosphamide'/exp OR 'cyclophosphamide':ab,ti OR 'cytophosphane':ab,ti OR 'cyclophosphamide monohydrate':ab,ti OR 'monohydrate, cyclophosphamide':ab,ti OR 'cyclophosphane':ab,ti OR 'cyclophosphamide anhydrous':ab,ti OR 'anhydrous, cyclophosphamide':ab,ti OR 'cytophosphan':ab,ti OR 'endoxan':ab,ti OR 'neosar':ab,ti OR 'NSC-26271':ab,ti OR 'NSC 26271':ab,ti OR 'NSC26271':ab,ti OR 'procytox':ab,ti OR 'sendoxan':ab,ti OR 'B-518':ab,ti OR 'B 518':ab,ti OR 'B518':ab,ti OR 'cytoxan':ab,ti OR 'cyclophosphamide':ab,ti

#3. Azathioprine

'azathioprine'/exp OR 'azathioprine':ab,ti OR 'imurel':ab,ti OR 'imuran':ab,ti OR 'imuran':ab,ti OR 'azathioprine sodium':ab,ti OR 'sodium, azathioprine':ab,ti OR 'azathioprine sodium salt':ab,ti OR 'azathioprine sulfate':ab,ti OR 'azathioprine':ab,ti

#4. Tacrolimus

'tacrolimus'/exp OR 'prograf':ab,ti OR 'prograf':ab,ti OR 'FR-900506':ab,ti OR 'FR 900506':ab,ti OR 'FR900506':ab,ti OR 'anhydrous tacrolimus':ab,ti OR 'tacrolimus, anhydrous':ab,ti OR 'tacrolimus anhydrous':ab,ti OR 'anhydrous, tacrolimus':ab,ti OR 'FK-506':ab,ti OR 'FK 506':ab,ti OR 'FK506':ab,ti OR 'tacrolimus':ab,ti

#5. Mycophenolic Acid

'mycophenolic acid'/exp OR 'mycophenolic acid':ab,ti OR 'mycophenolate mofetil':ab,ti OR 'mofetil, mycophenolate':ab,ti OR 'mycophenolic acid Morpholinoethyl Ester':ab,ti OR 'cellcept':ab,ti OR 'mycophenolate sodium':ab,ti OR 'sodium mycophenolate':ab,ti OR 'mycophenolate, sodium':ab,ti OR 'myfortic':ab,ti OR 'mycophenolate mofetil hydrochloride':ab,ti OR 'mofetil hydrochloride, mycophenolate':ab,ti OR 'RS 61443':ab,ti OR 'RS-61443':ab,ti OR 'RS61443':ab,ti

#6. Steroids

'glucocorticoid'/exp OR 'corticosteroid'/exp OR 'steroids'/exp OR 'dexamethasone'/exp OR ' prednisolone'/exp OR ' prednisone'/exp OR 'hydrocortisone'/exp OR 'cortisone'/exp OR 'budesonide'/exp OR 'beclomethasone'/exp OR 'glucocorticoid':ab,ti OR 'hormones, adrenal cortex':ab,ti OR 'corticosteroids':ab,ti OR 'corticoids':ab,ti OR 'methylfluorprednisolone':ab,ti OR 'hexadecadrol':ab,ti OR

'decameth':ab,ti OR 'decaspray':ab,ti OR 'dexasone':ab,ti OR 'dexpak':ab,ti OR 'maxidex':ab,ti OR 'millicorten':ab,ti OR 'oradexon':ab,ti OR 'decaject':ab,ti OR 'hexadrol':ab,ti OR 'dehydrocortisone':ab,ti OR 'delta-cortisone':ab,ti OR 'rectodelt':ab,ti OR 'prednison Hexal':ab,ti OR 'sterapred':ab,ti OR 'ultracorten':ab,ti OR 'winpred':ab,ti OR 'apo-prednisone':ab,ti OR 'cortan':ab,ti OR 'cortancyl':ab,ti OR 'panafcort':ab,ti OR 'cutason':ab,ti OR 'decortin':ab,ti OR 'dacortin':ab,ti OR 'decortisyl':ab,ti OR 'deltasone':ab,ti OR 'encortone':ab,ti OR 'encorton':ab,ti OR 'enkortolon':ab,ti OR 'kortancyl':ab,ti OR 'liquid pred':ab,ti OR 'meticorten':ab,ti OR 'orasone':ab,ti OR 'panasol':ab,ti OR 'predni tablinen':ab,ti OR 'prednidib':ab,ti OR 'predniment':ab,ti OR 'prednison Acsis':ab,ti OR 'acsis, prednison':ab,ti OR 'pronisone':ab,ti OR 'sone':ab,ti OR 'prednison Galen':ab,ti OR 'predate':ab,ti OR 'predonine':ab,ti OR 'Di-Adreson-F':ab,ti OR 'Di Adreson F':ab,ti OR 'DiAdresonF':ab,ti OR 'cortisone acetate':ab,ti OR 'cortone acetate':ab,ti OR 'adreson':ab,ti OR 'pulmicort':ab,ti OR 'rhinocort':ab,ti OR 'horacort':ab,ti OR 'beclometasone':ab,ti OR 'asmabec clickhaler':ab,ti OR 'ascocortonyl':ab,ti OR 'beclamet':ab,ti OR 'beclo asma':ab,ti OR 'beclo AZU':ab,ti OR 'beclocort':ab,ti OR 'beclomet':ab,ti OR 'bemedrex easyhaler':ab,ti OR 'beclomethasone dipropionate':ab,ti OR 'beclorhinol':ab,ti OR 'sanasthmax':ab,ti OR 'becloturmant':ab,ti OR 'beclovent':ab,ti OR 'beconase':ab,ti OR 'becloforte':ab,ti OR 'becodisk':ab,ti OR 'becodisks':ab,ti OR 'propaderm':ab,ti OR 'becotide':ab,ti OR 'sanasthmyl':ab,ti OR 'beconase AQ':ab,ti OR 'bronchocort':ab,ti OR 'junik':ab,ti OR 'ovar':ab,ti OR 'ecobec':ab,ti OR 'beclazone':ab,ti OR 'beclazone easy breathe':ab,ti OR 'ventolair':ab,ti OR 'prolair':ab,ti OR 'filair':ab,ti OR 'filair forte':ab,ti OR 'aerobec forte':ab,ti OR 'aerobec':ab,ti OR 'nasobec aqueous':ab,ti OR 'respocort':ab,ti OR 'vancenase':ab,ti OR 'vanceril':ab,ti OR 'aldecin':ab,ti OR 'viarin':ab,ti OR 'Apo-Beclomethasone':ab,ti

#1 AND (#2 OR #3 OR #4 OR #5 OR #6)

Cochrane Library search strategy

#1. IgA nephropathy

MeSH descriptor: [Glomerulonephritis, IGA] explode all trees

OR immunoglobulin A nephropathy:ti,ab,kw OR berger disease:ti,ab,kw OR glomerulonephritis, iga:ti,ab,kw OR IgA nephropathy:ti,ab,kw OR Glomerulonephritis, IGA:ti,ab,kw OR IgA nephritis:ti,ab,kw OR IgA glomerulonephritis:ti,ab,kw OR IgAN:ti,ab,kw OR Berger's disease:ti,ab,kw

#2. Cyclophosphamide

MeSH descriptor: [Cyclophosphamide] explode all trees

OR

cyclophosphamide:ti,ab,kw OR cyclophosphamide:ti,ab,kw OR cytophosphane:ti,ab,kw OR cyclophosphamide monohydrate:ti,ab,kw OR monohydrate, cyclophosphamide:ti,ab,kw OR cyclophosphane:ti,ab,kw OR cyclophosphamide anhydrous:ti,ab,kw OR anhydrous, cyclophosphamide:ti,ab,kw OR cytophosphan:ti,ab,kw OR endoxan:ti,ab,kw OR neosar:ti,ab,kw OR NSC-26271:ti,ab,kw OR NSC 26271:ti,ab,kw OR NSC26271:ti,ab,kw OR procytox:ti,ab,kw OR sendoxan:ti,ab,kw OR B-518:ti,ab,kw OR B 518:ti,ab,kw OR B518:ti,ab,kw OR cytoxan:ti,ab,kw OR cyclophosphamide:ti,ab,kw

#3. Azathioprine

MeSH descriptor: [Azathioprine] explode all trees

OR

azathioprine:ti,ab,kw OR azathioprine:ti,ab,kw OR imurel:ti,ab,kw OR imuran:ti,ab,kw OR imuran:ti,ab,kw OR azathioprine sodium:ti,ab,kw OR sodium, azathioprine:ti,ab,kw OR azathioprine sodium salt:ti,ab,kw OR azathioprine sulfate:ti,ab,kw OR azathioprine:ti,ab,kw

#4. Tacrolimus

MeSH descriptor: [Tacrolimus] explode all trees

OR

t acrolimus:ti,ab,kw OR prograf:ti,ab,kw OR prograf:ti,ab,kw OR FR-900506:ti,ab,kw OR FR 900506:ti,ab,kw OR FR900506:ti,ab,kw OR anhydrous tacrolimus:ti,ab,kw OR tacrolimus, anhydrous:ti,ab,kw OR tacrolimus anhydrous:ti,ab,kw OR anhydrous, tacrolimus:ti,ab,kw OR FK-506:ti,ab,kw OR FK 506:ti,ab,kw OR FK506:ti,ab,kw OR tacrolimus:ti,ab,kw

#5. Mycophenolic Acid

MeSH descriptor: [Mycophenolic Acid] explode all trees

OR

mycophenolic acid:ti,ab,kw OR mycophenolic acid:ti,ab,kw OR mycophenolate mofetil:ti,ab,kw OR mofetil, mycophenolate:ti,ab,kw OR mycophenolic acid Morpholinoethyl Ester:ti,ab,kw OR cellcept:ti,ab,kw OR mycophenolate sodium:ti,ab,kw OR sodium mycophenolate:ti,ab,kw OR mycophenolate, sodium:ti,ab,kw OR myfortic:ti,ab,kw OR mycophenolate mofetil hydrochloride:ti,ab,kw OR mofetil hydrochloride, mycophenolate:ti,ab,kw OR RS 61443:ti,ab,kw OR RS-61443:ti,ab,kw OR RS61443:ti,ab,kw

#6. Steroids

MeSH descriptor: [glucocorticoids] explode all trees OR MeSH descriptor: [Adrenal Cortex Hormones] explode all trees OR MeSH descriptor: [steroids] explode all trees OR MeSH descriptor: [dexamethasone] explode all trees OR MeSH descriptor: [prednisolone] explode all trees OR MeSH descriptor: [prednisone] explode all trees OR MeSH descriptor: [hydrocortisone] explode all trees OR MeSH descriptor: [cortisone] explode all trees OR MeSH descriptor: [budesonide] explode all trees OR MeSH descriptor: [beclomethasone] explode all trees

OR

glucocorticoid:ti,ab,kw OR hormones, adrenal cortex:ti,ab,kw OR corticosteroids:ti,ab,kw OR corticoids:ti,ab,kw OR methylfluorprednisolone:ti,ab,kw OR hexadecadrol:ti,ab,kw OR decameth:ti,ab,kw OR decaspray:ti,ab,kw OR dexasone:ti,ab,kw OR dexpak:ti,ab,kw OR maxidex:ti,ab,kw OR millicorten:ti,ab,kw OR oradexon:ti,ab,kw OR decaject:ti,ab,kw OR hexadrol:ti,ab,kw OR dehydrocortisone:ti,ab,kw OR delta-cortisone:ti,ab,kw OR rectodelt:ti,ab,kw OR prednison Hexal:ti,ab,kw OR sterapred:ti,ab,kw OR ultracorten:ti,ab,kw OR winpred:ti,ab,kw OR apo-prednisone:ti,ab,kw OR cortan:ti,ab,kw OR cortancyl:ti,ab,kw OR panafcort:ti,ab,kw OR cutason:ti,ab,kw OR decortin:ti,ab,kw OR dacortin:ti,ab,kw OR decortisyl:ti,ab,kw OR deltasone:ti,ab,kw OR encortone:ti,ab,kw OR encorton:ti,ab,kw OR enkortolon:ti,ab,kw OR kortancyl:ti,ab,kw OR liquid pred:ti,ab,kw OR meticorten:ti,ab,kw OR orasone:ti,ab,kw OR panasol:ti,ab,kw OR predni tablinen:ti,ab,kw OR prednidib:ti,ab,kw OR predniment:ti,ab,kw OR prednison Acsis:ti,ab,kw OR acsis, prednison:ti,ab,kw OR pronisone:ti,ab,kw OR sone:ti,ab,kw OR prednison Galen:ti,ab,kw OR predate:ti,ab,kw OR predonine:ti,ab,kw OR Di-Adreson-F:ti,ab,kw OR Di Adreson F:ti,ab,kw OR DiAdresonF:ti,ab,kw OR cortisone acetate:ti,ab,kw OR cortone acetate:ti,ab,kw OR adreson:ti,ab,kw OR pulmicort:ti,ab,kw OR rhinocort:ti,ab,kw OR horacort:ti,ab,kw OR beclometasone:ti,ab,kw OR asmabec clickhaler:ti,ab,kw OR ascocortonyl:ti,ab,kw OR beclamet:ti,ab,kw OR beclo asma:ti,ab,kw OR beclo AZU:ti,ab,kw OR beclocort:ti,ab,kw OR beclomet:ti,ab,kw OR bemedrex easyhaler:ti,ab,kw OR beclomethasone dipropionate:ti,ab,kw OR beclorhinol:ti,ab,kw OR sanasthmax:ti,ab,kw OR becloturmant:ti,ab,kw OR beclovent:ti,ab,kw OR beconase:ti,ab,kw OR

becloforte:ti,ab,kw OR becodisk:ti,ab,kw OR becodisks:ti,ab,kw OR propaderm:ti,ab,kw OR becotide:ti,ab,kw OR sanasthmyl:ti,ab,kw OR beconase AQ:ti,ab,kw OR bronchocort:ti,ab,kw OR junik:ti,ab,kw OR ovar:ti,ab,kw OR ecobec:ti,ab,kw OR beclazone:ti,ab,kw OR beclazone easy breathe:ti,ab,kw OR ventolair:ti,ab,kw OR prolair:ti,ab,kw OR filair:ti,ab,kw OR filair forte:ti,ab,kw OR aerobec forte:ti,ab,kw OR aerobec:ti,ab,kw OR nasobec aqueous:ti,ab,kw OR respocort:ti,ab,kw OR vancenase:ti,ab,kw OR vanceril:ti,ab,kw OR aldecin:ti,ab,kw OR viarin:ti,ab,kw OR Apo-Beclomethasone:ti,ab,kw

#1 AND (#2 OR #3 OR #4 OR #5 OR #6)

Table S2: Disposition after full text review

**Author**	**Journal**	**Decision**
Rauen 2015	N Engl J Med	EXCLUDE-Initial report of included study
Lai 1987	Br Med J	EXCLUDE- Didn't meet our requirements of treatment
Liu 2014	Intern Med.	EXCLUDE- Didn't meet our requirements of treatment
Xu 2014	Kaohsiung J Med Sci	EXCLUDE- Didn't meet our requirements of treatment; non-RCT
**Liu 2014**	**Int J Clin Pharmacol Ther**	**INCLUDE**
Cheng 2015	Nephrology	EXCLUDE- Didn't meet our requirements of treatment
Min 2017	Oncotarget	EXCLUDE- Didn't meet our requirements of treatment
Pozzi 2013	J Nephrol	EXCLUDE-Initial report of included study
**Hou 2017**	**Am J Kidney Dis**	**INCLUDE**
**Hogg 2015**	**Am J Kidney Dis**	**INCLUDE**
**Yu 2017**	**PLoS One**	**INCLUDE**
**Kim 2013**	**PLoS One**	**INCLUDE**
Liu 2010	Int J Clin Pharmacol Ther	EXCLUDE- Didn't meet our requirements of treatment
**Frisch 2005**	**Nephrol Dial Transplant**	**INCLUDE**
**Maes 2004**	**Kidney Int**	**INCLUDE**
Tang 2005	Kidney Int	EXCLUDE-Initial report of included study
**Rauen 2018**	**J Am Soc Nephrol**	**INCLUDE**
Lou 2006	Nephrology	EXCLUDE- Didn't meet our requirements of treatment

Woo 1988	Ann Acad Med Singapore	EXCLUDE-The outcome we needed was unobtained
Stangou 2011	Clin Exp Nephrol	EXCLUDE-The outcome we needed was unobtained
**Kamei 2011**	**Clin J Am Soc Nephrol**	**INCLUDE**
**Pozzi 2010**	**J Am Soc Nephrol**	**INCLUDE**
**Yoshikawa 2006**	**Clin J Am Soc Nephrol**	**INCLUDE**
Yoshikawa 1999	J Am Soc Nephrol	EXCLUDE-The outcome we needed was unobtained
**Tang 2010**	**Kidney Int**	**INCLUDE**
**Fellstrom 2017**	**Lancet**	**INCLUDE**
**Hogg 2006**	**Clin J Am Soc Nephrol**	**INCLUDE**
**Lv 2017**	**JAMA**	**INCLUDE**
**Manno 2009**	**Nephrol Dial Transplant**	**INCLUDE**
**Pozzi 2004**	**J Am Soc Nephrol.**	**INCLUDE**
**Katafuchi 2003**	**Am J Kidney Dis**	**INCLUDE**
**Lv 2009**	**Am J Kidney Dis**	**INCLUDE**
Koike 2008	Clin Exp Nephrol	EXCLUDE- The outcome we needed was unobtained
**Harmankaya 2002**	**Int Urol Nephrol**	**INCLUDE**
**Lai 1986**	**Clin Nephrol**	**INCLUDE**
**Shoji 2000**	**Am J Kidney Dis**	**INCLUDE**
**Julian 1993**	**Contrib Nephrol**	**INCLUDE**

Janki 2015	Transpl Int	EXCLUDE – Retrospective study
**Ballardie 2002**	**J Am Soc Nephrol**	**INCLUDE**
Walker 1990	Clin Nephrol	EXCLUDE- Didn't meet our requirements of treatment
Woo 1987	Clin Nephrol	EXCLUDE- Didn't meet our requirements of treatment
Yagi 2003	Clin Exp Nephrol	EXCLUDE- Didn't meet our requirements of treatment
Rasche 2016	Clin Exp Immunol	EXCLUDE- Didn't meet our requirements of treatment
Tang 2005	Kidney Int	EXCLUDE-Initial report of included study
Hogg 2004	BMC Nephrol	EXCLUDE-Initial report of included study
Locaelli 1999	J Nephrol	EXCLUDE- The outcome we needed was unobtained
Pozzi 2013	J Nephrol	EXCLUDE-Initial report of included study
Yoshikawa 1999	J Am Soc Nephrol	EXCLUDE-Initial report of included study

Table S3: **Assessment of risk of bias according to the Cochrane Risk of Bias tool for studies included in the network meta-analysis.**

	Random Sequence Generation (selection bias)	Allocation concealment (selection bias)	Blinding of participants and personnel (performance bias)	Blinding of outcome assessment (detection bias)	Incomplete outcome data (attrition bias)	Selective reporting (reporting bias)	Other bias
Fellstrom 2017	L	L	L	L	L	L	L
Hogg 2006	L	L	U	L	U	U	L
Lv 2017	L	L	L	L	L	L	L
Manno 2009	L	U	L	L	L	L	U
Pozzi 2004	L	U	H	U	H	U	L
Katafuchi 2003	U	U	U	U	H	L	U
Lv 2009	U	U	H	U	L	L	L
Harmankaya 2002	U	U	H	U	H	H	U
Lai 1986	U	U	H	U	L	L	U
Shoji 2000	L	U	H	U	U	H	H
Julian 1993	L	U	H	U	H	H	L
Liu 2014	L	L	U	L	U	L	L
Hou 2017	L	L	U	L	L	L	L
Hogg 2015	U	U	L	L	H	L	L
Yu 2017	L	L	L	L	H	L	L
Kim 2013	L	L	L	L	H	L	L
Frisch 2005	L	L	L	L	L	L	L
Maes 2004	U	U	U	L	L	L	L
Rauen 2018	U	U	U	L	L	L	L

Kamei 2011	U	U	U	L	L	L	L
Pozzi 2010	L	L	U	L	L	L	L
Yoshikawa 2006	L	L	H	L	L	L	L
Tang 2010	U	U	L	L	L	L	L
Ballardie 2002	U	U	H	L	L	L	L
L: Low risk; H: High risk; U: Unclear.							

Table S4. GRADE Quality of Evidence on the Efficacy and safety of immunosuppressive therapies in the Treatment of IgA nephropathy

Immunosuppressive therapies compared to supportive care as first-line therapy for IgA nephropathy				
**Patient or population:** Adult patients with IgAN<br>**Setting:** RCT<br>**Intervention:** Any immunosuppressive therapy<br>**Comparison:** Supportive care<br>**Outcomes:** Primary Outcome (deterioration of renal function)				
**Treatment**	**Relative effect (95% CI)**	**No. of Participants (Studies)**	**Quality of the evidence**	**Comments**
**Steroids**	**0.28 (0.12, 0.54)**	642 (12 RCTs)	⊕⊕⊕◯ Moderate	inconsistency
**Tacrolimus**	1.1 (0.14, 9.1)	18 (1 RCT)	⊕⊕⊕◯ Moderate	imprecision resulted from small sample
**Mycophenolate mofetil**	0.73 (0.22, 2.7)	100 (4 RCTs)	⊕⊕◯◯ Low	inconsistency from interventions and heterogeneity
**Cyclophosphamide**	2.2 (0.21, 28.0)	42 (1 RCT)	⊕⊕◯◯ Low	imprecision resulted from small sample and heterogeneity
**Azathioprine**	0.37 (0.069, 2.0)	122 (2 RCTs)	⊕⊕⊕◯ Moderate	inconsistency from interventions
**Cyclophosphamide+ Azathioprine**	0.58 (0.16, 2.8)	46 (2 RCTs)	⊕⊕◯◯ Low	imprecision resulted from small sample and heterogeneity

Immunosuppressive therapies compared to supportive care as first-line therapy for IgA nephropathy				
**Patient or population:** Adult patients with IgAN<br>**Setting:** RCT<br>**Intervention:** Any immunosuppressive therapy<br>**Comparison:** Supportive care<br>**Outcomes:** Complete remission				
**Treatment**	**Relative effect (95% CI)**	**No. of Participants (Studies)**	**Quality of the evidence**	**Comments**
**Steroids**	3.1 (0.53, 21.0)	160 (3 RCTs)	⊕⊕⊕○ Moderate	inconsistency
**Tacrolimus**	2.4 (0.27, 25.0)	38 (2 RCT)	⊕⊕⊕○ Moderate	imprecision resulted from small sample
**Mycophenolate mofetil**	2.2 (0.14, 16.0)	153 (3 RCTs)	⊕⊕⊕○ Moderate	inconsistency from interventions
**Cyclophosphamide**	1.2 (0.023, 26.0)	42 (1 RCT)	⊕⊕⊕○ Moderate	imprecision resulted from small sample
**Cyclophosphamide+ Azathioprine**	1.6 (0.079, 38.0)	27 (1 RCT)	⊕⊕⊕○ Moderate	imprecision resulted from small sample

Immunosuppressive therapies compared to supportive care as first-line therapy for IgA nephropathy				
**Patient or population:** Adult patients with IgAN<br>**Setting:** RCT<br>**Intervention:** Any immunosuppressive therapy<br>**Comparison:** Supportive care<br>**Outcomes:** Total remission				
**Treatment**	**Relative effect (95% CI)**	**No. of Participants (Studies)**	**Quality of the evidence**	**Comments**
**Steroids**	1.3 (0.79, 3.4)	241 (5 RCTs)	⊕⊕⊕◯ Moderate	inconsistency
**Tacrolimus**	3.1 (0.91, 12.0)	38 (2 RCT)	⊕⊕⊕◯ Moderate	imprecision resulted from small sample
**Mycophenolate mofetil**	1.2 (0.47, 3.5)	170 (4 RCTs)	⊕⊕⊕◯ Moderate	inconsistency from interventions
**Cyclophosphamide**	0.84 (0.17, 5.1)	42 (1 RCT)	⊕⊕⊕◯ Low	imprecision resulted from small sample
**Cyclophosphamide+ Azathioprine**	1.6 (018, 18.0)	27 (1 RCT)	⊕⊕⊕◯ Moderate	imprecision resulted from small sample

Immunosuppressive therapies compared to supportive care as first-line therapy for IgA nephropathy				
**Patient or population:** Adult patients with IgAN<br>**Setting:** RCT<br>**Intervention:** Any immunosuppressive therapy<br>**Comparison:** Supportive care<br>**Outcomes:** Serious adverse effects				
**Treatment**	**Relative effect (95% CI)**	**No. of Participants (Studies)**	**Quality of the evidence**	**Comments**
**Steroids**	1.1 (0.37, 2.5)	608 (8 RCTs)	⊕⊕○○ Low	Inconsistency and heterogeneity
**Tacrolimus**	3.0 (0.31, 37.0)	20 (1 RCT)	⊕⊕⊕○ Moderate	imprecision resulted from small sample
**Mycophenolate mofetil**	0.4. (0.086, 1.5)	211 (6 RCTs)	⊕⊕○○ Low	inconsistency from interventions and heterogeneity
**Cyclophosphamide**	0.17 (0.0026, 4.7)	42 (1 RCTs)	⊕⊕○○ Low	imprecision resulted from small sample and heterogeneity
**Azathioprine**	0.53 (0.049, 3.7)	122 (2 RCTs)	⊕⊕⊕○ Moderate	inconsistency from interventions
**Cyclophosphamide+ Azathioprine**	2.5 (0.39, 17.0)	27 (1 RCT)	⊕⊕⊕○ Moderate	imprecision resulted from small sample

Figure S5. Characteristics of pathology in each included study. T, treatment group. C, control. NA, not available. M, mesangial proliferation. E, endocapillary proliferation. S, segmental glomerulosclerosis. T, tubular atrophy or interstitial fibrosis.

Study	Characteristics of Pathology	
	T	C
**Fellstrom 2017**	NA	
**Hogg 2006**	26±22% endocapillary hypercellularity<br>5±6% cellular or fibrocellular crescents<br>9±9% fibrotic crescents<br>23±14% segmental glomerulosclerosis<br>1.4±0.9% interstitial fibrosis	44±25% endocapillary hypercellularity<br>9±9% cellular or fibrocellular crescents<br>13±15% fibrotic crescents<br>30±18% segmental glomerulosclerosis<br>1.2±0.7% interstitial fibrosis
**Lv 2017**	57.6% patients with M<br>31.6% patients with E<br>71.2% patients with S<br>61.3% patients with T<br>58.6% patients with C	61.0% patients with M<br>23.8% patients with E<br>72.4% patients with S<br>65.0% patients with T<br>43.0% patients with C
**Manno 2009**	Histological grade G2 (moderate) lesions	
**Pozzi 2004**	53% histologic score mild<br>35% histologic score moderate<br>12% histologic score severe	60% histologic score mild<br>35% histologic score moderate<br>5% histologic score severe
**Katafuchi 2003**	Glomerular score 5.6±1.1	Glomerular score 5.4±1.1
**Lv 2009**	Histological score 5 (1-11)	Histological score 6 (1-15)
**Harmankaya 2002**	100% patients with M<br>4.8% patients with glomerulosclerosis<br>4.8% patients with tubular atrophy	100% patients with M<br>9.1% patients with glomerulosclerosis<br>4.5% patients with tubular atrophy
**Lai 1986**	41.2% Histological grade G1<br>41.2% Histological grade G2<br>17.6% Histological grade G3	23.5% Histological grade G1<br>41.2% Histological grade G2<br>35.3% Histological grade G3
**Shoji 2000**	65.0±27.2% mesangial cell proliferation<br>7.6±5.5% cellular crescents<br>2.3±5.1% fibrotic crescents<br>7.9±6.1% segmental sclerosis<br>0.82±0.4% interstitial fibrosis	59.3±14.7% mesangial cell proliferation<br>1.9±3.6% cellular crescents<br>0±0% fibrotic crescents<br>4.1±7.9% segmental sclerosis<br>0.75±0.46% interstitial fibrosis
**Julian 1993**	NA	
**Liu 2014**	Lee III or Lee IV	
**Hou 2017**	At least 1 of the following histologic lesions:<br>cellular and fibrocellular crescents involving 10% to <50% of glomeruli, endocapillary hypercellularity, or glomerular necrosis, with additional inclusion criteria being tubular atrophy/interstitial fibrosis involving <50%.	
**Hogg 2015**	59% patients with M<br>73% patients with E<br>91% patients with S<br>41% patients with T	42% patients with M<br>83% patients with E<br>92% patients with S<br>21% patients with T

**Yu 2017**	NA	
**Kim 2013**	64.7% patients with M<br>11.8% patients with E<br>70.6% patients with S<br>17.6% patients with T	31.3% patients with M<br>37.5% patients with E<br>87.5% patients with S<br>31.2% patients with T
**Frisch 2005**	presence of glomerulosclerosis, tubulointerstitial fibrosis and/or crescent formation in 25% of the biopsy sample.	
**Maes 2004**	Histologic unfavorable criteria (grades II to IV defined by Churg and Sobin and/or glomerular capillary wall IgA deposits	
**Rauen 2018**	NA	
**Kamei 2011**	5.7±8.6% sclerosis<br>23.6±20.7% crescents<br>9.6±10.0% capsular adhesions<br>91.7±7.4% mesangial proliferations	4.1±5.8% sclerosis<br>21.2±17.9% crescents<br>7.0±7.1% capsular adhesions<br>90.2±7.0% mesangial proliferations
**Pozzi 2010**	NA	
**Yoshikawa 2006**	3.9±8.0% sclerosis<br>17.8±18.0% crescents<br>5.5±8.2% capsular adhesions	2.7±5.0% sclerosis<br>19.3±17.1% crescents<br>3.9±5.2% capsular adhesions
**Tang 2010**	Morphologic score II、II、IV and absence of crescent	
**Ballardie 2002**	mesangial proliferative glomerulonephritis with focal endocapillary proliferation and focal segmental and global glomerulosclerosis. Interstitial inflammation and fibrosis were present while crescents were absent.	

The original data of this meta-analysis can be searched at
https://osf.io/t98zp/?view_only=0462d8bcb84b4ccbb1f87e94fe279dcf
